# Validation of Hv_1_ channel functions in BV2 microglial cells using small molecule modulators

**DOI:** 10.3389/fncel.2025.1624224

**Published:** 2025-07-29

**Authors:** Ashutosh Sharma, Nandini B. Kale, Priyanka Yadav, Shivani Yadav, Madhavi Ranawat, Valmik S. Shinde, Aravind Singh Kshatri

**Affiliations:** ^1^Neuroscience and Ageing Biology Division, CSIR-Central Drug Research Institute (CDRI), Lucknow, India; ^2^Academy of Scientific and Innovative Research (AcSIR), Ghaziabad, India; ^3^Medicinal and Process Chemistry Division, CSIR-CDRI, Lucknow, India

**Keywords:** validation, microglia, Hv1 channels, neuroinflammation, activator, inhibitor, signalling pathway, ROS

## Abstract

Microglia are the first responders to insults or damages in the brain where they display both beneficial and detrimental effects. Excessively activated microglia aggravate the secondary damage by producing several proinflammatory mediators. Voltage-gated proton channels, Hv_1_ are selectively expressed in the microglia where they modulate microglial activation. Therefore, Hv_1_ has emerged as a tractable target for treating a number of conditions, ranging from pain, neurological disorders to cancer. Due to the absence of a suitable Hv_1_ inhibitor, the pathophysiological roles of Hv_1_ channels has been exemplified using preclinical Hv_1_ knockout (KO) mice models. Thus, we characterized and validated the microglial Hv_1_ channel’s functions using the recently reported Hv_1_ inhibitor (YHV98-4) and a novel Hv_1_ activator (S-023-0515) in a model of lipopolysaccharide (LPS)-induced neuroinflammation. In LPS-stimulated BV2 microglial cells, treatment with YHV98-4 alleviated the proinflammatory cytokines such as TNF-α, IL-6, and iNOS. Direct activation of Hv_1_ channels using S-023-0515 resulted in an increase in microglial M1 like polarisation, proinflammatory mediators, phagocytic capacity and mitochondrial ROS levels but did not alter the cellular ROS production. Analysis of the signalling pathway indicated that YHV98-4 and S-023-0515 exerted their protective and deleterious effects, respectively via phosphorylation of NF-κΒ, which serves as an upstream regulator of the inflammatory cascade. Collectively, our results elucidate the essential role of Hv_1_ channels in microglial functions and also demonstrate that their pharmacological inhibition and activation during inflammatory conditions are neuroprotective or neurotoxic, respectively.

## Graphical abstract

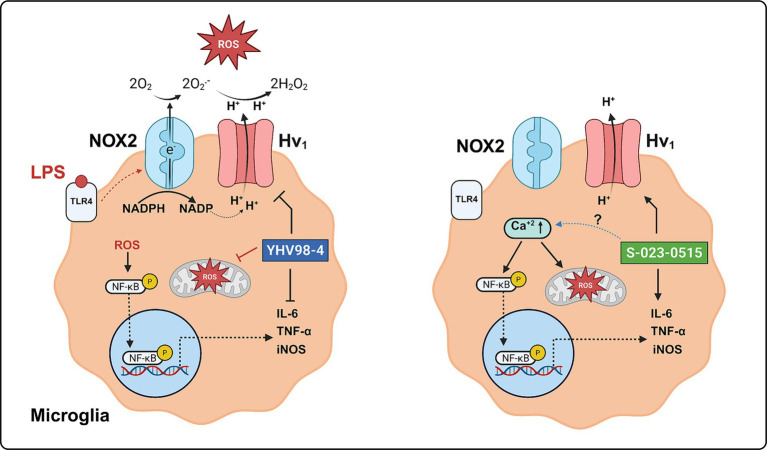

## 1 Introduction

Chronic inflammation is a common clinical characteristic shared by the majority of neurodegenerative diseases, including multiple sclerosis, Alzheimer’s disease, Parkinson’s disease, and amyotrophic lateral sclerosis (Zhang et al., 2023). In the central nervous system, neuroinflammation is choreographed by non-neuronal cells such as microglia and astrocytes. Upon a brain insult/immunological challenge, microglia gets activated to secrete various cytotoxic factors and proinflammatory mediators, including reactive oxygen species (ROS) and cytokines such as IL-1β, IL-6, iNOS, and TNFα (Muzio et al., 2021). Sustained release of these neurotoxic factors from overactivated microglia results in neuronal dysfunction/death, resulting in the pathogenesis of neurodegenerative disorders (Dheen et al., 2007). Therefore, attenuating microglia-mediated inflammatory responses represents an attractive strategy to counteract brain degeneration in neurodegenerative disorders.

Voltage-gated proton channel (Hv_1_) is a homodimer with each subunit containing a proton conducting voltage sensor domain (VSD; Ramsey et al., 2006). Two gated proton permeation pathways of native Hv_1_ channels mediate the H^+^ efflux in the phagocytes of the immune system (Ramsey et al., 2009) and microglia of the central nervous system (Wu et al., 2012). These channels extrude protons from the phagocytes/microglia to compensate for charge and osmotic imbalances, which are critical for sustaining the NADPH Oxidase 2 (NOX2) activity. Thus, Hv_1_ activity results in an increased driving force for extracellular Ca^2+^ entry, sustained NOX2 activity, energy consumption and microglial activation. The resulting activated microglia produce pro-inflammatory cytokines, reactive oxygen species (ROS) and nitric oxide (NO), which damage neurons, leading to neuroinflammation and subsequent neurodegeneration. Hv_1_ activity during microglial activation exacerbates neuroinflammation into the injured/aberrant microenvironment during ischemic stroke (Wu et al., 2012), traumatic brain injury (Ritzel et al., 2021), spinal cord injury (Li et al., 2021), chronic pain (Zhang et al., 2022) and neurodegenerative conditions such as multiple sclerosis (Chen et al., 2020) and Parkinson’s disease (Neal et al., 2023). Using the transgenic mice models lacking Hv_1_ channels (Hv_1_^−/−^), all these studies have demonstrated the beneficial neuroprotective effects such as dampened microglial activation, decreased production of proinflammatory cytokines, reduced acidosis and ROS levels and diminished neuronal death. Therefore, Hv_1_ channels represent an ideal target for reducing microglial activation during neuroinflammation.

To date, the molecular mechanisms of Hv_1_ channel activation, pathways of proton efflux, and the pharmacological tools that can assist in understanding the physiological role of Hv_1_ channels still need to be discovered. In the past decade, numerous compounds have been identified via virtual and high-throughput screening assays as Hv_1_ inhibitors, but none have displayed good selectivity and an acceptable pharmacokinetic profile *in vivo*. Pharmacologically, these channels can be inhibited by Zn^2 + 12^, ClGBI (Hong et al., 2014) and a peptide inhibitor (Corza6, C6; Zhao et al., 2018). Zn^2+^ is the classical inhibitor for Hv_1_ channels (IC_50_ = 2 μM) but its therapeutic usage is limited because of its involvement in numerous cellular processes (Qiu et al., 2016). Guanidine derivatives, including ClGBI, are well-known Hv_1_ channel inhibitors (IC_50_ = 26 μM), but they lack selectivity, potentially leading to off-target effects (Szanto et al., 2023). C6 is a designer peptide that specifically blocks Hv_1_ with an IC_50_ of 30 nM, but its therapeutic applications are questionable due to its undefined pharmacokinetics (plasma stability, membrane permeability, circulation half-life, etc.; Zhao et al., 2018; Zhao et al., 2022). Currently, there are no small molecules that are known to activate Hv_1_ channels. Arachidonic acid and albumin are known to induce an increase in the amplitude of Hv_1_ proton currents with an EC_50_ of 7 μM and 75 μM, respectively (Henderson et al., 1997; Suszták et al., 1997; Cherny et al., 2001; Zhao et al., 2021; Kawanabe and Okamura, 2016; Han et al., 2023). Recently, a small molecule, YHV98-4, has been identified as a selective Hv_1_ inhibitor with an IC_50_ of ~ 1 μM (Zhang et al., 2022). This compound reduced the intracellular alkalization, ROS production and pro-inflammatory chemokine release in chronic pain mice models to alleviate inflammatory pain. Although the primary functions of Hv_1_ channels in the animal models are well established using gene knockout strategy, its precise roles *in vitro* are not clearly defined using pharmacological tools. Therefore, we have characterized and validated the microglial functions of Hv_1_ channels using the previously reported inhibitor YHV98-4 and a novel activator S-023-0515, which is an analogue of YHV98-4. Our results unequivocally support the previous *in vivo data* of Hv_1_ channel deletion/suppression in neuroinflammation is indeed neuroprotective. Additionally, we also provide evidence that direct activation of microglial Hv_1_ channels can trigger inflammation and neurotoxicity. The observed toxic effects (increase in M1 polarization, proinflammatory cytokines, phagocytosis and mitochondrial ROS) occurred in a NOX2-independent manner, highlighting a non-NOX/ROS dependent Hv_1_ mediated inflammatory pathway in the microglia.

## 2 Results

### 2.1 Expression of Hv_1_ channels in BV2 microglial cells

**Figure 1:**
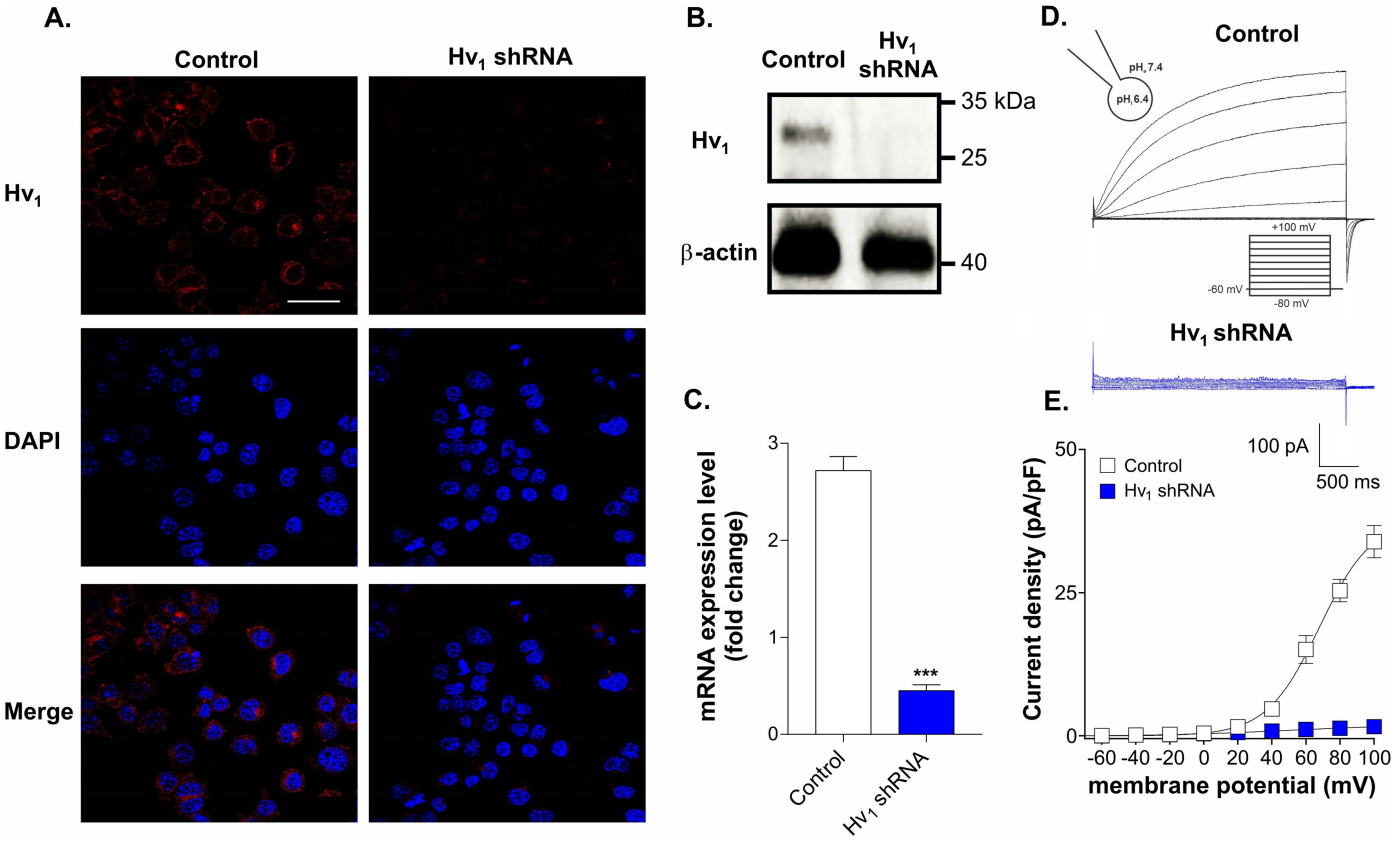
Expression of Hv_1_ channels in BV2 microglial cells. **(A)** Representative immunocytochemistry images of scramble shRNA transduced cells (control) and Hv_1_ shRNA transduced cells stained with anti-Hv_1_ antibody (red) and DAPI (blue). Scale bar = 40 μM **(B)** A comparison of Hv_1_ channel protein expression by western blot in control cells and in Hv_1_ shRNA transduced cells. **(C)** Hv_1_ mRNA expression in control and Hv_1_ shRNA cells was detected by using RT-PCR. **(D)** Representative whole-cell currents recorded in response to 3 s long pulses, stepping from a holding potential of −60 mV to levels ranging from −80 to +100 mV, with 20 mV increments in control (black traces) and Hv_1_ shRNA cells (blue traces). **(E)** Mean current density versus membrane potential curves corresponding to the control condition and Hv_1_ shRNA condition is plotted. Current density was measured by normalizing maximal peak currents with cell capacitance (pA/pF). ***p < 0.001, two-tailed unpaired t-test, *N* = 3.

To confirm the expression of Hv_1_ channels in BV2 microglial cells ICC, western blot, RT-PCR and patch clamp electrophysiology techniques were used. As shown in Figures 1A–C, the surface expression, mRNA expression levels and protein levels of Hv_1_ channels were clearly detected in BV2 cells. Next, we have knocked down (KD) the Hv_1_ gene using shRNA lentiviral particles. Immunofluorescence assay, western blotting and RT-PCR results confirmed that the expression of Hv_1_ protein was significantly suppressed in BV2 cells transduced with the Hv_1_ shRNA group compared to the control shRNA. To validate these results we recorded Hv_1_ channel currents in both wild type cells and KD cells using the whole-cell configuration of the patch clamp technique. The endogenous proton currents were elicited by depolarising voltage steps of 3 s from a holding potential of −60 mV every 10 s. Under an acidic intracellular solution (pH_i_ at 6.4, pH_e_ at 7.4, ΔpH = 1), slowly activating, outwardly rectifying Hv_1_ currents were observed in the BV2 cells (Figure 1D, black traces), which were practically abolished in the Hv_1_ shRNA transduced cells (blue traces). The resultant current densities (pA/pF) were plotted in Figure 1E where Hv_1_ currents were observed at voltages exceeding +20 mV in control group but not in the Hv_1_ shRNA group. Together, these results confirm the presence of Hv_1_ channels in BV2 cells.

### 2.2 Identification of a novel Hv_1_ channel activator

**Figure 2:**
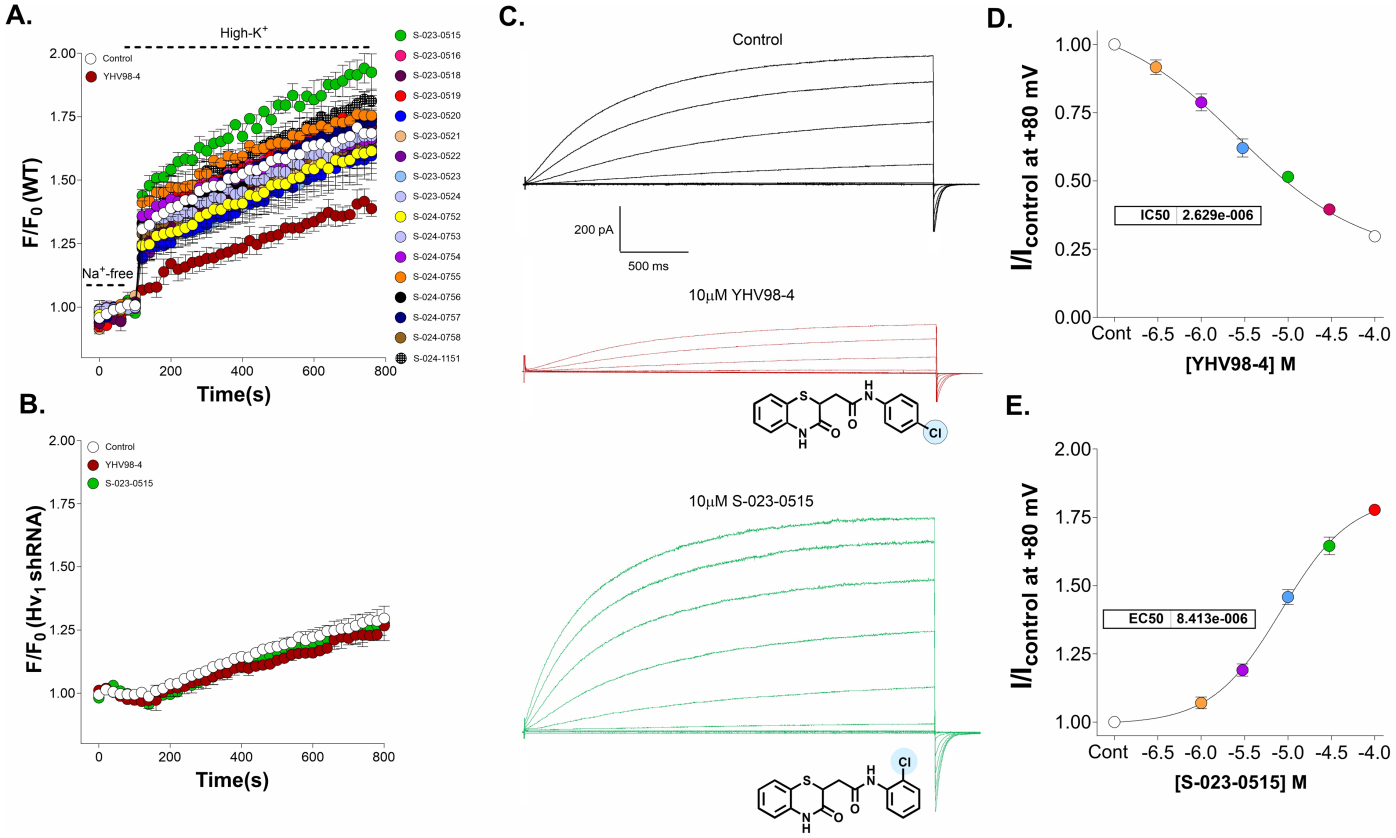
Identification of a novel Hv_1_ channel activator. **(A)** Primary screening of novel chemical entities using BCECF revealed that S-023-0515 (green circles) is an activator of Hv_1_ channels. YHV98-4 (brown circles) is used as a reference standard. All compounds were tested at a concentration of 10 μM. **(B)** Knockdown of Hv_1_ ablated both the stimulatory and inhibitory effects of S-023-0515 and YHV98-4, respectively. **(C)** Whole-cell current recordings of Hv_1_ channels in the absence (control, black traces) and presence of 10 μM YHV98-4 (brown traces and 10 μM S-023-0515 (green traces). Structures of the compounds are shown inset. **(D,E)** Normalized currents (I/I_control_) for each drug concentration at +80 mV were fitted to the four-parameter hill equation to yield IC50 values of 2.6 ± 1.1 μM (*N* = 4–6) for YHV98-4 **(D)** and EC50 of 8.41 ± 1.9 μM (*N* = 4–6) for S-023-0515 **(E)**.

To identify Hv_1_ channel modulators, we have developed a screening assay using pH-sensitive probe BCECF. Its fluorescence intensity at maximum emission wavelength is pH dependent: a fall in pH causes a decrease in fluorescence intensity, and a rise in pH increases its fluorescence intensity. BV2 microglial cells were acid-preloaded by the “rebound acidification” technique using 30 mM NH_4_Cl (Boron and De Weer, 1976), and the normalized fluorescence change caused by pH recovery with high K^+^ solution was measured. The involvement of Na^+^/H^+^ exchanger in pH recovery is excluded by applying a washout step with Na^+^ free solution (NMDG) before depolarization induced by high K^+^ solution (150 mM). Under this condition, the intracellular pH rapidly recovered upon stimulation with high K^+,^ which can be visualized as an increase in the normalised fluorescence (F/F_0_) of BCECF dye (Figure 2A, white circles). Using this assay, a library of 17 compounds (at a final concentration of 10 μM) belonging to the YHV98-4 series (structures are shown in Supplementary Figure S1) were screened. As reported earlier (Zhang et al., 2022), the application of YHV98-4 (chloro group in para position; brown circles) dramatically reduced the pH recovery (fluorescence reduced by 47 ± 3.1% compared to control at t = 600 s), highlighting the inhibitory effect of this compound on Hv_1_ channels. Although no compounds were better than YHV98-4 in inhibiting the Hv_1_ channels, S-023-0515 (chloro group in ortho position; green circles) increased the BCECF fluorescence by >30%, suggesting that it could be a potential activator of the Hv_1_ channels. In addition to this compound, a modest Hv_1_ potentiating effect was also seen in cells treated with S-024-1151 (fluoro group in ortho position; cyan circles) and S-024-0755 (methyl group in ortho position; orange circles). To rule out the possibility of this effect not being a fluorescence artefact, we have used the Hv_1_ shRNA cells and performed the fluorescent assay. When these cells were acid-loaded and depolarised, the pH recovery was found to be significantly reduced compared to the WT cells (Figure 2B). Additionally, both the inhibitory and stimulatory effects of YHV98-4 and S-023-0515, respectively, were abolished, confirming the target specificity of these compounds. Additionally, the possible biological targets of these molecules, which were assessed using the PASS online server. The top predicted direct targets for YHV98-4 and S-023-0515 are presented in Supplementary Tables 1, 2, respectively. None of the ion channels were found to be top targets for these molecules, hinting at a specificity for Hv_1_ channels. To determine the maximal concentration of these compounds that the cells could tolerate without observable cell death, an MTT assay was performed (Supplementary Figure S2). A 10 μM concentration of these molecules had no significant toxic effect on BV2 cells after 24 h treatment. Further, we have established the effects of these compounds directly on Hv_1_ channel currents in BV2 cells. Application of 10 μM YHV98-4 inhibited the currents as previously reported (Zhang et al., 2022), whereas 10 μM S-023-0515 increased the Hv_1_ channel currents (panel 2C). Sequential application of increasing concentrations of YHV98-4 and S-023-0515 produced a concentration-dependent block and enhancement of Hv_1_ currents at a single voltage step to +80 mV, respectively, which were normalized and fitted with Hill equation to yield an IC_50_ of 2.6 ± 1.1 μM (*N* = 4–6 cells, Figure 2D) and EC_50_ of 8.41 ± 1.9 μM (*N* = 4–6 cells, Figure 2E).

### 2.3 Microglial M1/M2 polarization is regulated by Hv_1_ channel activity

**Figure 3:**
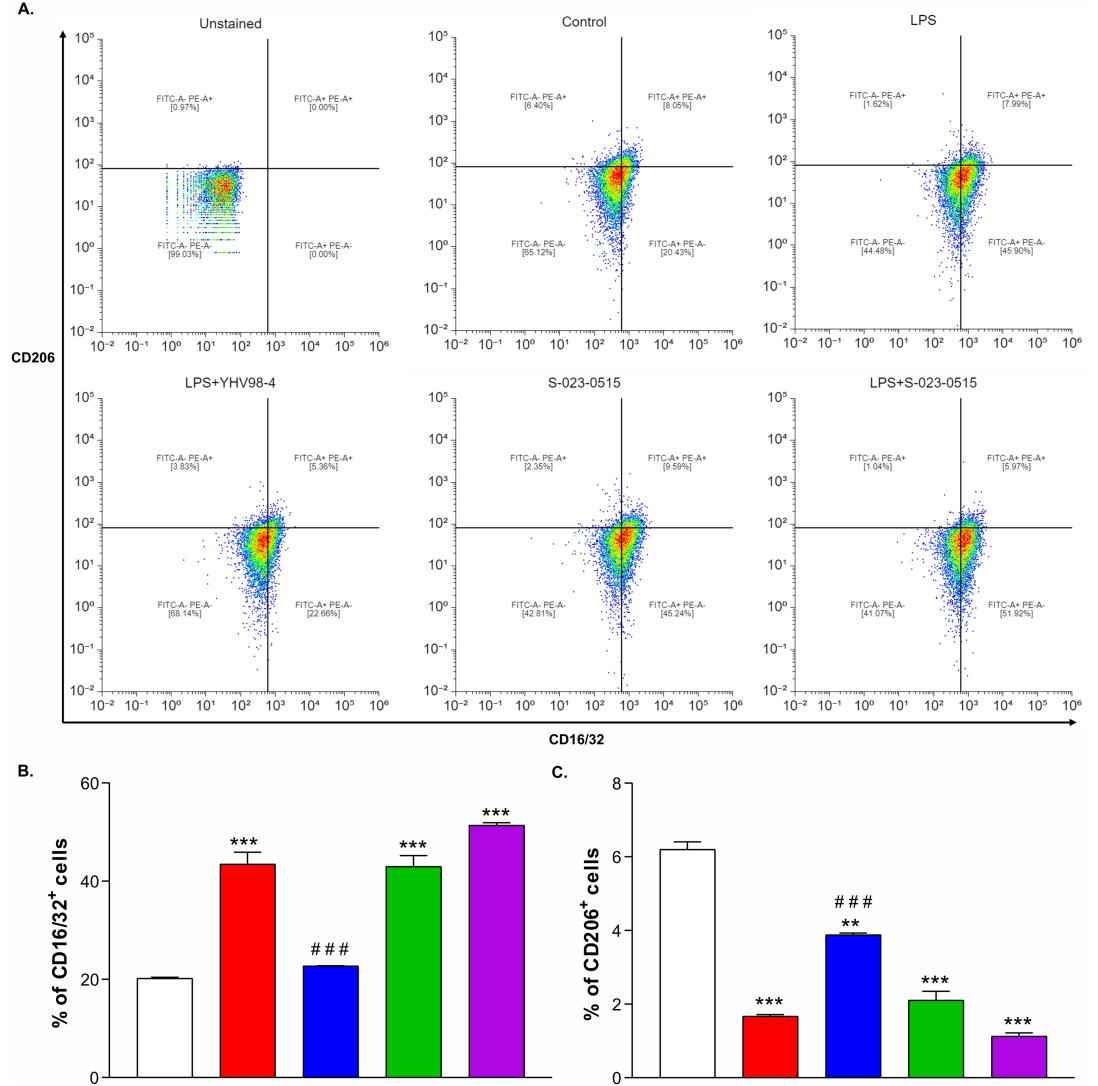
Direct Hv_1_ channel activation promoted microglia towards proinflammatory phenotype. **(A-F)** Representative flow cytometry plots showing the expression patterns CD16/32/FITC (M1 marker) and CD206/PE (M2 marker) in unstained cells **(A)**, Control cells **(B)**, LPS **(C)**, LPS + YHV98-4 **(D)**, S-023-0515 **(E)** and LPS + S-023-0515 **(F)**. 0.1% DMSO as Control, 100 ng/ml of LPS and 10 μM concentration of the compounds were used for 24 h treatment. (G, H) The mean % of CD16/32^+^ cells and CD206^+^ cells for each condition is quantified and shown. All the data are expressed as means ± SEM and were analysed using ordinary one-way ANOVA followed by Tukey’s *post hoc* test. *N* = 3 independent experiments. *p < 0.05, **p < 0.01, ***p < 0.001 compared to control. ^#^*p* < 0.05, ^##^*p* < 0.01, ^###^*p* < 0.001 compared to LPS.

Microglia M1/M2 polarization is a key factor regulating the neuroinflammatory responses. Therefore, flow cytometry was performed to assess the microglial polarization phenotype induced by YHV98-4 and S-023-0515. CD16/32 was used as a classical M1 phenotype (pro-inflammatory) cell surface marker, whereas CD206 was used as an M2 phenotype (anti-inflammatory) cell surface marker. When the cells were treated with LPS, the percentage of CD16/32^+^ cells were significantly increased from 20.2 ± 0.2% to 43.5 ± 2.4% (Figures 3A,B), and this increase was blunted upon application of YHV98-4 (22.7 ± 0.1%, Figure 3B). This beneficial effect of YHV98-4 was paralleled by a partial increase in CD206^+^ cells (from 6.2 ± 0.2% to 3.9 ± 0.2%, Figure 3C). Intriguingly, direct activation of Hv_1_ channels using S-023-0515 favoured the M1 phenotype cells (42.9 ± 2.2%, Figure 3B) and also diminished the M2 polarized cells (2.1 ± 0.2%, Figure 3C). These results suggested that the activation of Hv_1_ channels with S-023-0515 treatment promoted pro-inflammatory microglia similar to LPS treatment, whereas the inhibition of Hv_1_ channels with YHV98-4 modulated microglia towards an anti-inflammatory phenotype.

### 2.4 Activation of Hv_1_ channels increased the expression of pro-inflammatory mediators

**Figure 4:**
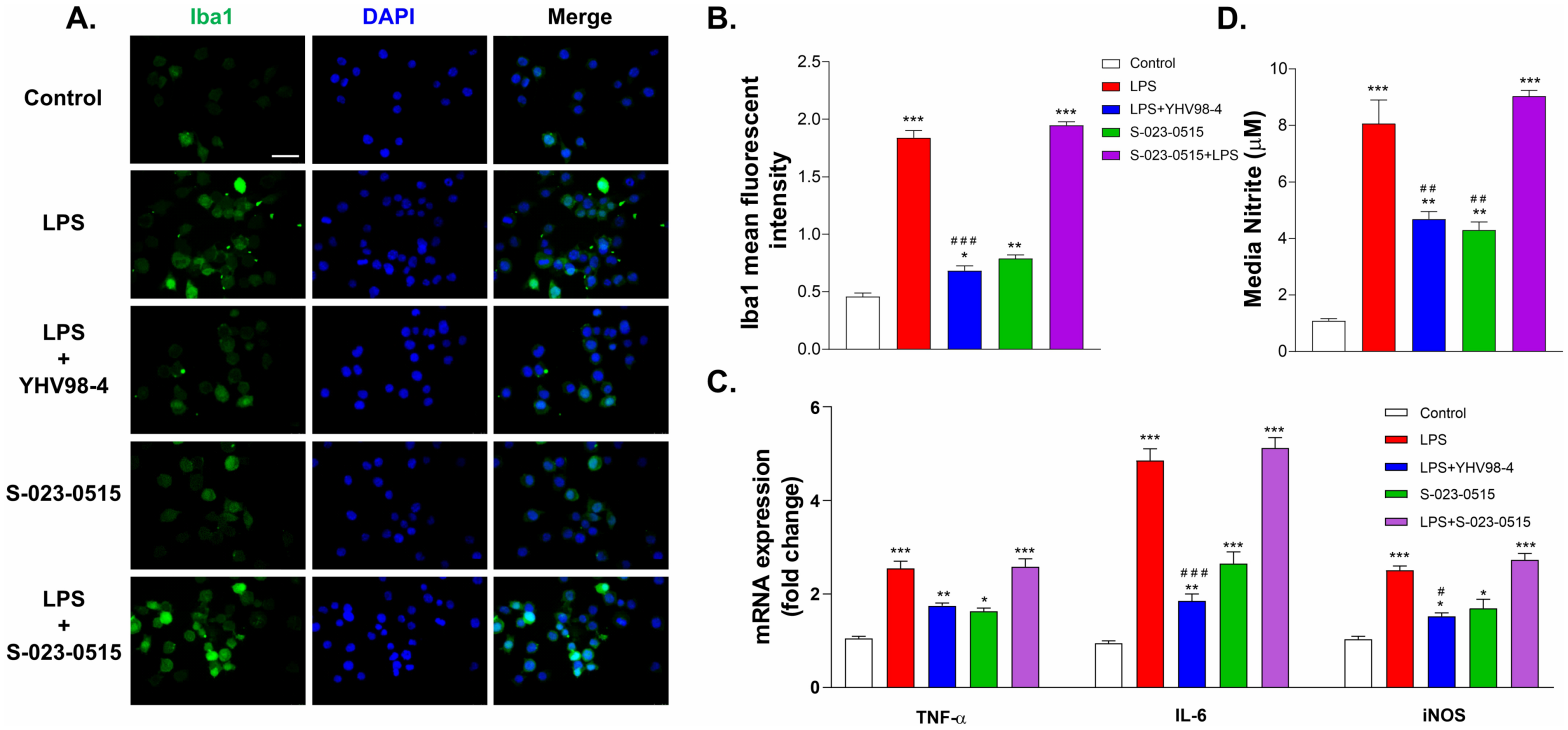
Hv_1_ channels mediate the release of proinflammatory cytokines from activated microglia. **(A,B)** Immunostaining and intensity analysis of Iba1 immunoreactivity of microglia after 24 h exposure with vehicle (control), LPS, LPS + YHV98-4, S-023-0515 and LPS + S-023-0515. Scale bar = 100 μM (C) The changes in mRNA expression levels of proinflammatory cytokines of these cells were quantified using RT-PCR. (D) Nitrite levels were determined for the above treatment groups using Greiss assay. All the data are expressed as means ± SEM and were analysed using ordinary one-way ANOVA followed by Dunnet’s post hoc test. *N* = 5 independent experiments. *p < 0.05, **p < 0.01, ***p < 0.001 compared to control. ^#^*p* < 0.05, ^##^*p* < 0.01, ^###^*p* < 0.001 compared to LPS.

Cytokines are well known to play crucial roles in manifestations of inflammation, and microglial activation results in the production of proinflammatory cytokines such as IL-6, TNF-α, iNOS, etc. (Smith et al., 2012). Therefore, we first established the microglial activation by immuno-labelling with Iba1 (ionized calcium-binding adapter molecule 1). LPS exposure for 24 h remarkably activated microglia as indicated by increased immunoreactivity and mean fluorescence intensity (Figures 4AB). YHV98-4 treatment significantly mitigated the LPS-induced expression of Iba1. As compared to the control, S-023-0515 also significantly upregulated the Iba1 expression levels (*p* < 0.001). Subsequently, we assessed the impact of Hv_1_ channel modulation on the levels of some important proinflammatory cytokines. LPS stimulation significantly increased the mRNA levels of IL-6, TNF-α and iNOS in BV2 cells, and such an increased mRNA level was partially reversed by treatment with 10 μM YHV98-4 (Figure 4C). Intriguingly, the levels of these cytokines were also significantly enhanced by treatment with 10 μM S-023-0515, but the combined treatment with LPS and the compounds did not increase the mRNA levels further. Additionally, the levels of NO were found to be increased after LPS stimulation (7.5 ± 1.3 μM in LPS vs. 1.1 ± 0.1 μM in control) and S-023-0515 treatment (2.9 ± 0.1 μM). However, pretreament with YHV98-4 attenuated the LPS-induced NO production (4.6 ± 0.3 μM, Figure 4D). These results confirm that LPS or S-023-0515 triggers microglial activation, resulting in an increased production of pro-inflammatory cytokines and NO production, which are largely suppressed by Hv_1_ antagonism.

### 2.5 S-023-0515 enhanced the microglial phagocytosis and mitochondrial ROS levels but not NOX2 dependent cellular ROS production

**Figure 5:**
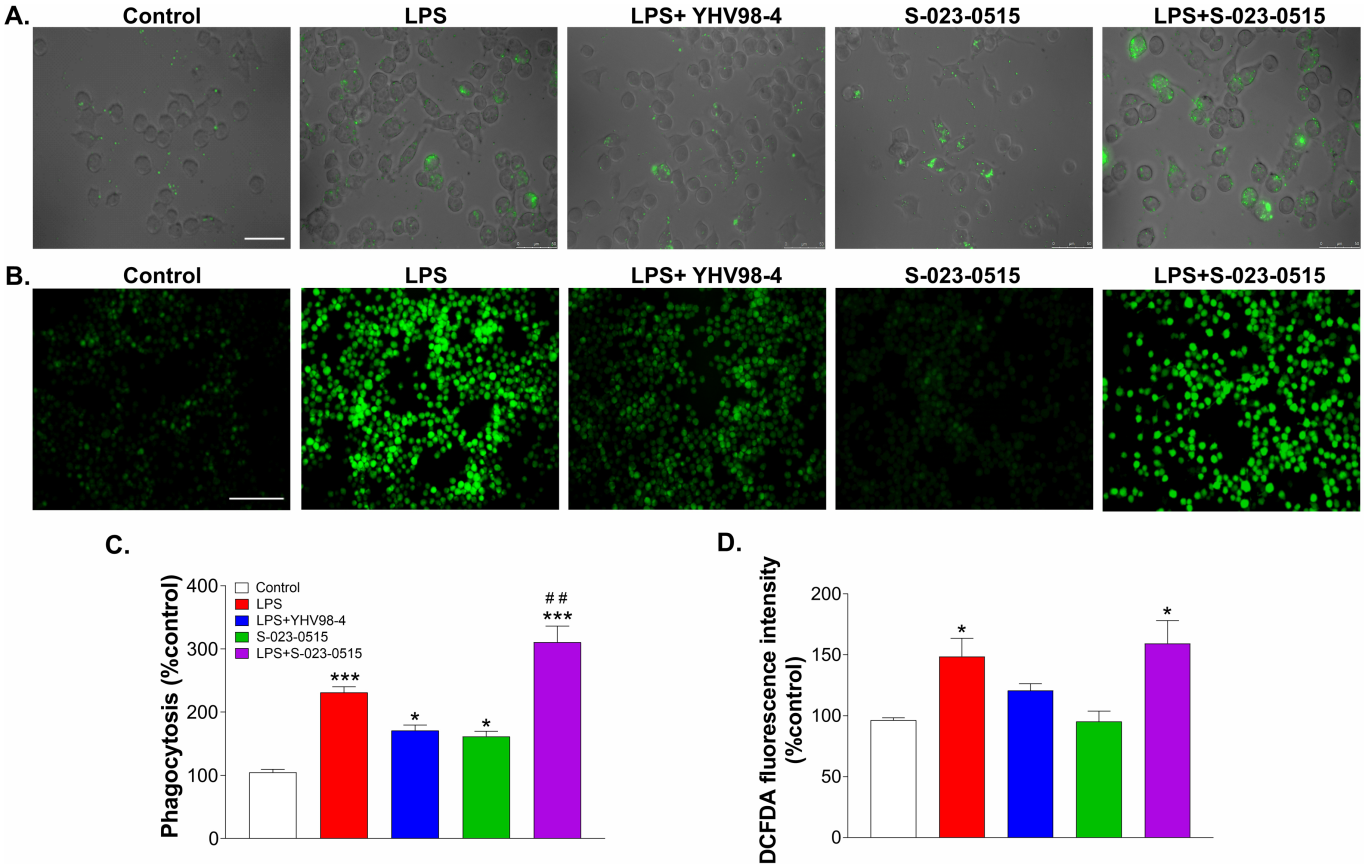
Microglial phagocytosis but not cellular ROS production is directly linked to the activity of Hv_1_ channels. **(A)** BV2 cells were incubated with FITC-conjugated latex beads that were pre-treated with vehicle (control), LPS, LPS + YHV98-4, S-023-0515 and LPS + S-023-0515 for 24 h and their phagocytic activity was observed by fluorescent microscopy. Scale bar = 100 μM. **(B)** Cells were pre-treated in all the above-mentioned conditions for 24 h, and the production of intracellular ROS was determined using H2DCFDA staining. Scale bar = 150 μM. **(C)** Mean phagocytosis (% control) for each treatment group is plotted. **(D)** The mean fluorescence intensity with respect to control for each group is quantified and shown. All the data are expressed as means ± SEM and were analysed using ordinary one-way ANOVA followed by Tukey’s post hoc test. *N* = 6 independent experiments. *p < 0.05, **p < 0.01, ***p < 0.001 compared to control. ^##^*p* < 0.01 compared to LPS.

**Figure 6:**
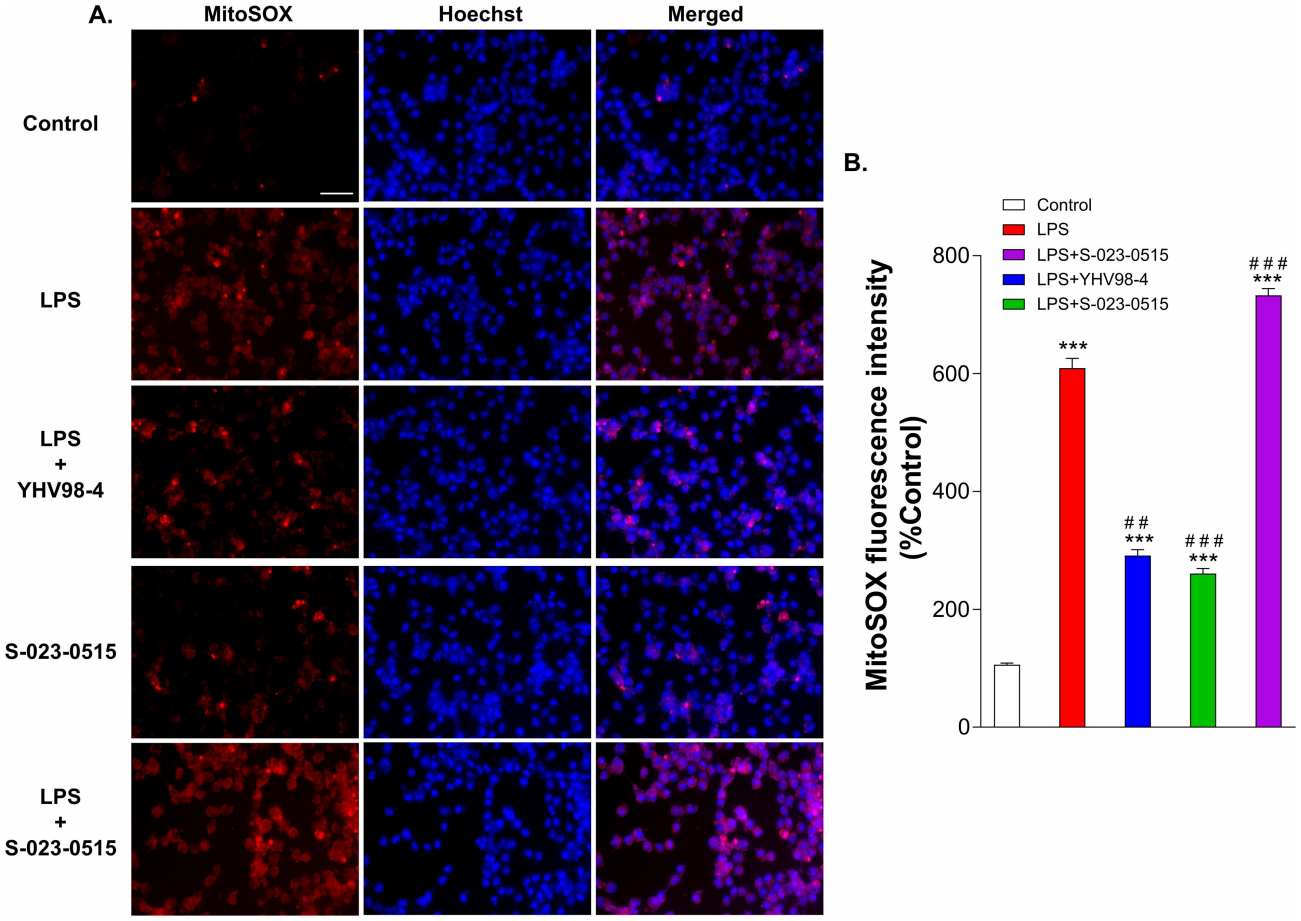
S-023-0515 treatment enhanced the mitochondrial superoxide production of BV2 cells. **(A)** Cells were pre-treated with vehicle (control), LPS, LPS + YHV98-4, S-023-0515 and LPS + S-023-0515 for 24 h and MitoSOX red staining was performed to detect the level of mitochondrial ROS. Hoechst 33342 was used to visualize nuclei. Scale bar = 50 μM. **(B)** The mean fluorescence intensity with respect to control for each group is quantified and shown. All the data are expressed as means ± SEM and were analysed using ordinary one-way ANOVA followed by Tukey’s post hoc test. *N* = 5 independent experiments. *p < 0.05, **p < 0.01, ***p < 0.001 compared to control. ^##^*p* < 0.01 compared to LPS.

Phagocytosis is one of the main functional aspects of microglia that help maintain proper neuronal circuit development and homeostasis (Galloway et al., 2019). Thus, we have tested the involvement of Hv_1_ channels in microglia phagocytosis using fluorescence-labeled latex beads. Before phagocytosis, BV2 cells were challenged with LPS, LPS + YHV98-4, S-023-0515, and LPS + S-023-0515 for 24 h (Figure 5A). In LPS treated cells, a significant number of latex beads were engulfed by cells compared to the control cells (*p* < 0.001, Figures 5A,C). Treatment with YHV98-4 in the presence of LPS partially reversed the phagocytosis of the beads. However, Hv_1_ channel activation with S-023-0515 enhanced the phagocytic ability of BV2 cells compared to control (*p* < 0.05), and this effect became additive post-treatment with LPS + S-023-0515 (*p* < 0.001). Microglia are one of the primary ROS-producing cells in the CNS, which require NOX2 activation for NOX2-dependent ROS generation (Haslund-Vinding et al., 2017). Another well-documented function of Hv_1_ channels in microglia is to sustain the activity of NOX2 for the production of NOX2-dependent ROS (Ramsey et al., 2009). Therefore, we have further investigated the Hv_1_-mediated effects of S-023-0515 on the overall cellular ROS and mitochondrial ROS levels using H2DCFDA and MitoSOX dyes, respectively. The levels of intracellular ROS were substantially increased following treatment with LPS, which is evident by a rise in H2DCFDA fluorescence (Figure 5B). Inhibiting the Hv_1_ channels with YHV98-4 effectively attenuated the levels of ROS released by LPS. Conversely, treatment with either S-023-0515 alone or in combination with LPS did not induce any further cellular ROS generation (Figure 5D). We further analysed the effects of the following treatments on the mitochondrial ROS generation. LPS treatment induced a significant increment (5.8-fold, *p* < 0.001, Figures 6A,B) in the mitoSOX red fluorescence compared to the control. Inhibition of Hv1 channels by YHV98-4 suppressed the LPS-induced mitochondrial ROS generation. Intriguingly, treatment with S-023-0515 resulted in a 2.5-fold increase in mitochondrial ROS levels compared to the control (*p* < 0.001, Figure 6). This data indicated that NOX2-independent activation of Hv_1_ channels leads to the induction of mitochondrial ROS but not the intracellular ROS.

### 2.6 Hv_1_ channel expression is unaltered after treatment with S-023-0515

**Figure 7:**
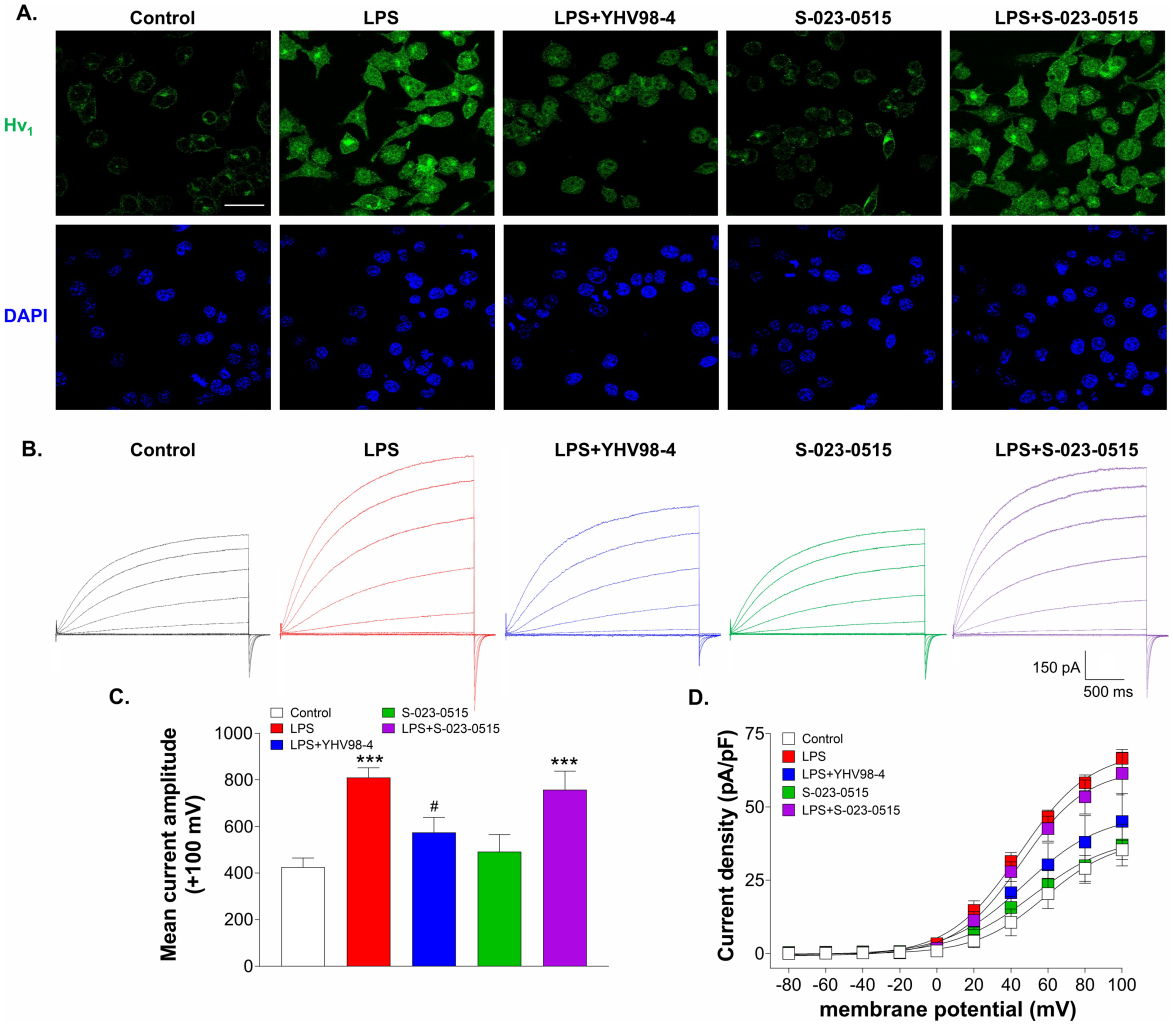
YHV98-4 blunted the LPS-induced upregulation of Hv_1_ channel current density. **(A)** Representative immunofluorescent images of Hv_1_ (green) protein level and DAPI (blue) in BV2 cells treated with vehicle (control), LPS, LPS + YHV98-4, S-023-0515 and LPS + S-023-0515 for 24 h. Scale bar = 10 μM. **(B)**Representative whole-cell recordings of BV2 cells that were exposed to vehicle (control, black traces), LPS (red traces), LPS + YHV98-4 (blue traces), S-023-0515 (green traces) and LPS + S-023-0515 (purple traces) for 24 h. **(C)** Mean current amplitude at +100 mV for each treatment group is plotted. **(D)** Mean current density (pA/pF) of each treatment group is plotted as a function of membrane potential. All the data are expressed as means ± SEM and were analysed using ordinary one-way ANOVA followed by Tukey’s post hoc test. *n* = 6–8 cells/group, *N* = 3 independent experiment for ICC and *N* = 5 independent experiments. ***p < 0.001 compared to control. ^#^*p* < 0.05 compared to LPS.

Inflammation is reported to upregulate the expression levels of Hv_1_ channels in both the central (Wu et al., 2012; Neal et al., 2023; Liu et al., 2015) and peripheral nervous systems (Zhang et al., 2022). Hence, we have determined whether treatment with S-023-0515 alters the surface expression levels of Hv_1_ channels in BV2 microglial cells. Firstly, LPS treatment of 100 ng/ml for 24 h increased the membrane expression levels of Hv_1_ channels (Figure 7A). This effect is paralleled by an increase in Hv_1_ channel current density by approximately 88% (red traces, Figure 7B) compared to the control (black traces). Interestingly, YHV98-4 treatment (10 μM) along with LPS (blue traces) diminished the LPS mediated enhancement of Hv_1_ expression and current density (mean current density: 45.1 ± 9 pF vs. 66.5 ± 3 pF in LPS at +100 mV). No significant changes in the Hv_1_ currents were observed when the cells were treated with 10 μM S-023-0515 (green traces) with respect to the control. Lastly, the combined treatment of LPS with S-023-0515 did not produce any additional enhancement of Hv_1_ currents compared to LPS (Figure 7B, purple traces).

### 2.7 Stimulation of NF-κΒ nuclear translocation by S-023-0515

**Figure 8:**
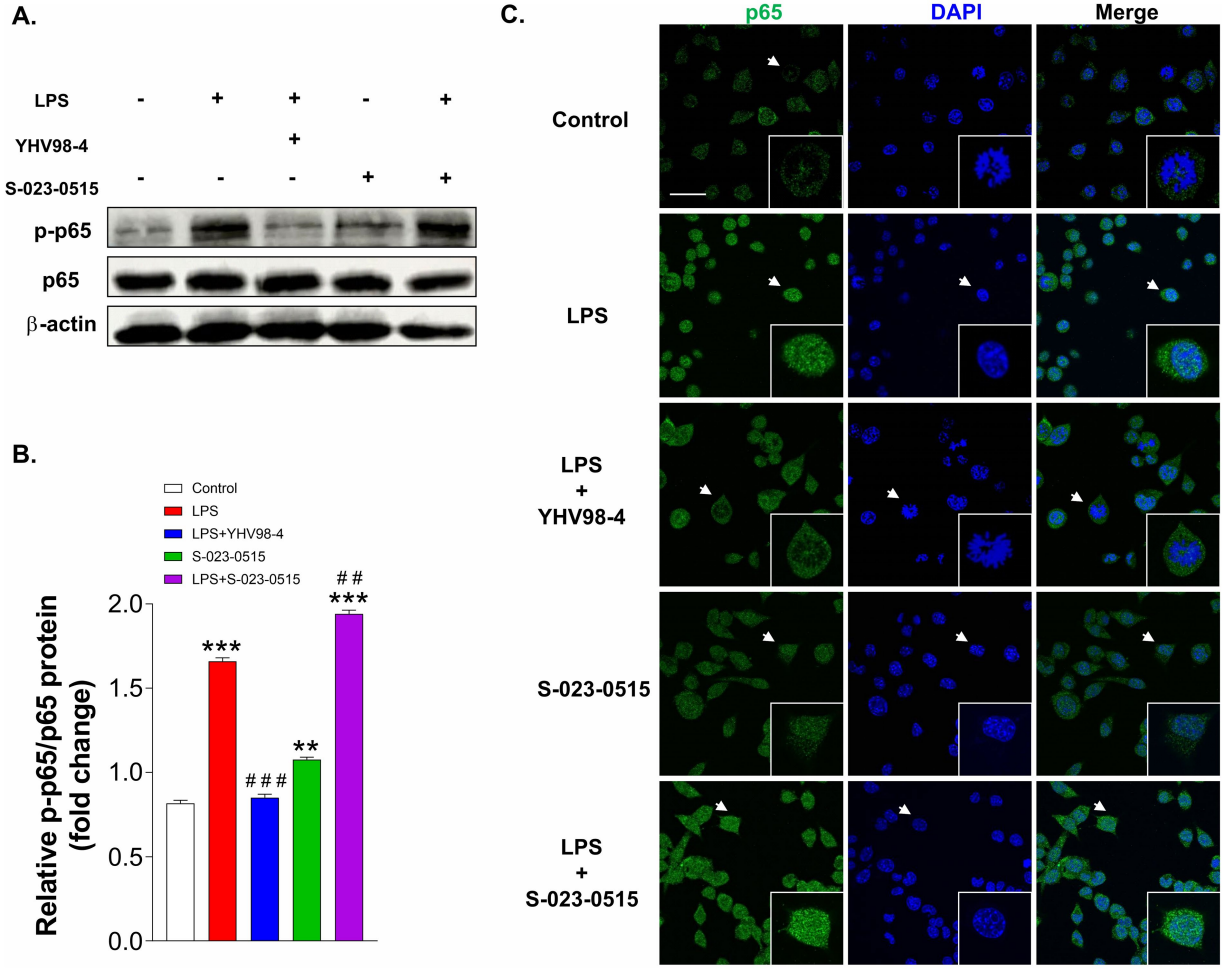
YHV98-4 and S-023-0515 differentially regulate the NF-κΒ pathway. **(A)** Western blot analysis of p-p65 and p65 proteins in microglia that were treated with vehicle (control), LPS, LPS + YHV98-4, S-023-0515 and LPS + S-023-051. **(B)** Densitometric analysis showing the quantification of p-p65 signal intensity that was normalized to p65. **(C)** Localization of p65 was visualized by fluorescence microscopy following immunofluorescence staining with an anti p65 antibody (green). The cells were also stained with DAPI to visualize nuclei (blue). Scale bar = 10 μM. White arrows shows the cells that are magnified at 4x in insets. All the data are expressed as means ± SEM and were analysed using ordinary one-way ANOVA followed by Tukey’s post hoc test. *N* = 3 independent experiments. **p < 0.01, ***p < 0.001 compared to control. ^##^*p* < 0.01, ^###^*p* < 0.001 compared to LPS.

The transcription factor, nuclear factor kappa-B (NF-κΒ) is a pivotal regulator of cytokine release, microglial polarization and ROS generation during inflammation (Liu et al., 2017; Bachstetter et al., 2011). Subsequently, we explored whether this signalling molecule role is involved in the pro- or anti-inflammatory effects of S-023-0515 and YHV98-4, respectively. Phospho-p65 (p-p65) is typically considered as an indicator of NF-κΒ activation (Zhong et al., 2002). Immunoblotting data using the total protein extracts revealed that pre-treatment with YHV98-4 mitigated the expression levels of p-p65 subunits in LPS-stimulated BV2 cells (Figures 8A,B). In contrast, S-023-0515 pre-treatment enhanced the phosphorylation of p65. Consistent with these results, our immunocytochemical analysis data also substantiated the finding that YHV98-4 prevented the translocation of the p65 subunit from the cytoplasm to the nucleus after LPS stimulation (Figure 8C). On the contrary, S-023-0515 potentiated the p65 translocation into the nucleus. These results demonstrate that microglial Hv_1_ channels modulate the NF-κΒ activity to manifest a pro- or anti-inflammatory response.

## 3 Discussion

Intracellular pH regulation is one of the tightly regulated processes which is governed by a dynamic interplay between transporters and ion channels. During inflammation, protons play a crucial role in microglial activation, reactive oxygen species production, phagocytic activity, as well the release of cytokines from microglia (Liu et al., 2010). Microglial Hv_1_ channels are known to be the first responders for NOX-dependent generation of ROS and intracellular pH regulation. Most of the previous work done on these channels demonstrated the beneficial neuroprotective effects *in vivo* using knockout animal models. Among the numerous potential Hv_1_ inhibitors described earlier, only YHV98-4 is identified as a potent inhibitor with good selectivity and an acceptable pharmacokinetic profile *in vivo* (Zhang et al., 2022). Here, we characterized and validated the functions of microglial Hv_1_ channels using a prototypical inhibitor (YHV98-4) and a novel activator (S-023-0515). In agreement with earlier studies, we also provided evidence *in vitro* that suppressing Hv_1_ channels impairs LPS-induced neuroinflammation. In contrast, direct activation of Hv_1_ channels independent of NOX2 stimulation: (1) Stimulated microglia and polarized them towards M1 like phenotype (2) Facilitated the production of pro-inflammatory cytokines (3) Enhanced phagocytic activity (4) Increased the mitochondrial ROS (5) Unchanged the intracellular ROS. (5) Activated the NF-κΒ signalling pathway.

The most surprising finding in our study is that a simple substitution of the chloro group from para to ortho position in the parent compound, YHV98-4, produced contrasting effects. Zhang et al. reported that this chloro group is essential for the increased affinity and potency of YHV98-4. Molecular docking and mutagenesis experiments in their study indicated that this compound binds in a cavity formed by the S0 segment and the intracellular ends of the four transmembrane segments (Zhang et al., 2022). Although the precise molecular mechanisms of Hv_1_ inhibition by YHV98-4 are not well established, we hypothesise that YHV98-4 prevents the movement of the voltage sensor domains and interferes with the proton conduction pathway. Likewise, S-023-0515 might bind in the same cavity as YHV98-4 but with different amino acid residues that may facilitate the movement of voltage sensors to activate the channel. More structural studies involving computational molecular dynamic simulations and crystal structures in apo- and ligand-bound states are needed to validate the underlying molecular mechanisms of action of these compounds on Hv_1_ channels. Our in-silico structure–activity study predicted that there is a low probability of binding of these two molecules with other ion channels. However, the Hv_1_ channel was also not predicted as a direct target for these compounds due to the lack of biologically active small molecules data against Hv_1_ in CHEMBEL database. To reinforce this selectivity data, the compound’s effect should be directly tested on various microglial ion channels and transporters.

Our electrophysiological analysis of Hv_1_ channel currents and molecular biology data, where the mRNA transcript of Hv_1_ was identified using RT-qPCR along with the Hv_1_ protein in western blots and immunostaining, confirms the abundant expression of Hv_1_ channels in the BV2 cell line. Two observations in our study support the previous conclusions that Hv_1_ channels contribute to the pH_i_ recovery. Firstly, inhibiting the Hv_1_ channels with YHV98-4 significantly slowed down the pH_i_ recovery (Figure 2A). Secondly, silencing of Hv_1_ channels drastically attenuated the recovery upon stimulation (Figure 2B). In this scenario, an alternative pH recovery mechanism involving Cl^−^ (Cl^−^/OH^−^) may also be possible, but earlier reports demonstrated that their contribution is minimal in these cells (Murphy et al., 2005). Together, our data reiterates previous studies (Morihata et al., 2000) that Hv_1_ channels are the primary pathway for proton extrusion and pH homeostasis in the microglia.

Apart from the quiescent state, activated microglia are traditionally characterised into two functionally distinct states, the classically activated M1 and the alternatively activated M2 (Tang and Le, 2016). M1-type microglia (marker C16/32) exhibit strong phagocytic ability and can produce several proinflammatory factors, including IL-1β, IL-6, TNF-α, and iNOS (Wang et al., 2015), thereby promoting inflammatory response and aggravating neuronal damage. M2-type microglia (marker CD206) displays a neuroprotective role by upregulating arginase 1, secreting growth factors and releasing anti-inflammatory cytokines such as IL-10 and TGF-β (Colonna and Butovsky, 2017). We demonstrated that YHV98-4 increased protein expression of CD206 and reduced the levels M1 related cytokines TNF-α, IL-6, and iNOS. Conversely, S-023-0515 caused the upregulation of CD16/32 cells and simultaneously increased the levels of M1 cytokines. A possible explanation for this effect is that mitochondrial ROS is known to drive the proinflammatory cytokine production (Naik and Dixit, 2011; Park et al., 2015). Our observation that S-023-0515 mediated a rise in mitochondrial ROS could have provoked the upregulation of proinflammatory cytokines. Nevertheless, these results should be validated further by using a mitochondrial ROS scavenger such as mito-TEMPO, N-acetyl-cysteine, etc. and investigating the contribution of mitochondrial ROS in proinflammatory responses. Future work should also include the assessment of M2 markers (such as Arg1, Ym1, IL-10, TGF-β) at a mRNA level and/or protein level to fully understand the effects of these molecules on microglial polarization. Together, our results indicate that Hv_1_ channel modulation is a key signalling pathway associated in the microglial M1 ➔ M2 like phenotype switching.

Oxidative stress mediated by ROS release from microglia promotes neuroinflammation. Microglial ROS are generated primarily by the activation of NOX2 (Kim et al., 2010) and secondarily by mitochondria (Simpson and Oliver, 2020). H2DCFDA and mitoSOX are commonly used fluorescent probes for detecting overall cellular ROS levels (Hempel et al., 1999) and mitochondria-specific ROS levels (Dikalov and Harrison, 2014), respectively. The role of Hv_1_ channels in the NOX2-mediated ROS production has been extensively studied earlier. An underlying mechanism is that NOX2 activation causes the depolarisation of the intracellular membrane due to the transfer of electrons from NADPH, and simultaneously, NADPH oxidation also generates free protons (H^+^), leading to cytosol acidification. These two signals trigger Hv_1_ channel activation to counteract these charge imbalances and sustain the activity of NOX2 for ROS production. In accordance with this mechanism, blocking of Hv_1_ channels inhibited NOX2-mediated ROS generation (DeCoursey et al., 2003). Previous studies have reported that the LPS-induced inflammatory response in microglia is directly associated with increased ROS production (both cellular and mitochondrial; Simpson and Oliver, 2020; Park et al., 2015) and that inhibition of the inflammatory response is associated with blocking ROS production (Iizumi et al., 2016; Sun et al., 2016; Kim et al., 2017). Our findings corroborated the previous observation that Hv_1_ inhibition suppressed the cellular ROS production in LPS-stimulated microglia. However, when the Hv_1_ channels are activated with S-023-0515, we did not observe an increase in total ROS production as evident by the fluorescence changes of H2DCFDA dye. One plausible explanation for this result is that NOX2 activity is shown to modulate the activation of Hv_1_ channels (Ramsey et al., 2009), but conversely, direct Hv_1_ stimulation might not induce NOX2 activation to result in increased cellular ROS production. Notably, we detected a significant rise in the mitochondrial ROS production after S-023-0515 treatment. A simplified explanation of these results is that the abnormal Ca^2+^ signalling triggered by cytosolic alkalinization led to excessive ROS generation by mitochondria. The interplay between the Ca^2+^ overload and mitochondrial ROS production is firmly established (Adam-Vizi and Starkov, 2010) and known to play a critical role in microglial dysfunction in the context of neurodegenerative diseases (Lin et al., 2022; Calvo-Rodriguez et al., 2020). A recent study identified that Hv_1_ channels localised in the mitochondria of medullary thick ascending limb (mTAL) cells of the kidney also produced mitochondrial ROS independent of NOX mediated ROS (Patel et al., 2019). The contribution of Hv_1_ channels in the regulation of cellular ROS and mitochondrial ROS warrants a future study. Experiments, such as quantification of NOX2 expression, gene knockdown studies, assessment of lipid peroxidation, measurement of intracellular ATP and mitochondrial membrane potential, would assist us in understanding Hv_1_ channels’ functions in NOX2-independent settings. Alternatively, employing NOX2 inhibitors such as DPI, apocynin and using extracellular specific ROS probes such as AmplexRed will shed more information on the NOX2-dependent mechanisms. Nonetheless, our data provide an indication that Hv_1_ channels may also function independently of NOX2 activation in the microglia, similar to that of sperm cells, medullary thick ascending limb (mTAL) cells of the kidney, snail neurons, basophils and osteoclasts (Seredenina et al., 2015; Patel et al., 2019).

Our data also showed that the Hv_1_ channel density is significantly increased upon LPS treatment, which is a key phenotypic change in microglia observed during the inflammatory insult (Li et al., 2021; Neal et al., 2023; Zhang et al., 2022). The precise mechanisms underlying these changes in proton channel expression upon inflammation have not yet been identified, but activated microglia are known to upregulate many other ion channels. For instance, proinflammatory microglia were shown to upregulate inward rectifier potassium (Kir) channels (Deftu et al., 2018), transient receptor potential channel 6 (TRPC6; Liu et al., 2017), and voltage-gated potassium channel 1.3 (K_v_1.3; Nguyen et al., 2017) expression. ROS are one of the key signal transducing molecules in neuroinflammation (Hsieh and Yang, 2013). It is previously shown to directly induce post-translational modifications of ion channels or indirectly alter the channel functions by affecting their transcription, trafficking and turnover signalling pathways (Annunziato et al., 2002; Liu and Gutterman, 2002). We speculate that ROS generated by LPS treatment may have upregulated Hv_1_ channel expression and its inhibition by YHV98-4 dampened the ROS levels to eventually lessen the Hv_1_ current density. In the case of S-023-0515, no apparent changes in ROS levels as well as Hv_1_ expression were observed, suggesting a ROS-dependent protein expression. Nevertheless, further studies at the molecular level are required to confirm this hypothesis.

NF-κΒ signalling pathway regulates the synthesis of proinflammatory mediators and cytokines during inflammation. In the unstimulated cells, NF-κΒ, a heterodimer consisting of p65 and p50 subunits, is localized to the cytosol. Upon external stimulus, NF-κΒ gets phosphorylated and translocated into the nucleus, where it induces several proinflammatory gene expressions (Hayden and Ghosh, 2004). Our data demonstrated that YHV98-4 suppressed the LPS-induced nuclear translocation of NF-κΒ p65, which is an indication of its anti-inflammatory effect. This result is in line with the previous observation that Hv_1_ deletion attenuated the phosphorylation of NF-κB p65 in a model of ischemic stroke (Wu et al., 2012). It may also be possible that YHV98-4 might be directly interfering with the other molecules in the LPS-induced NF-κΒ signalling cascade (e.g., TLR4, MyD88, TIRF etc.) and exerted its protective effects. Interestingly, after S-023-0515 treatment, a prominent p-p65 band in the western blot, as well as enhanced nuclear p65 staining, were observed, indicating Hv_1_ channel activation results in the induction of NF-κΒ pathway. A possible explanation for this finding is that the constitutive Hv_1_ channel activity due to S-023-0515 binding may have caused alkalization-induced Ca^2+^ influx to phosphorylate p65 of the NF-κΒ pathway (Ca^2+^/NF-kΒ pathway). Alternatively, S-023-0515 produced a non-specific effect on microglial calcium channels resulting in an influx of Ca^2+^. Earlier work also suggested that the key functions of microglia (such as motility, ramification cytokine release, phagocytosis and receptor trafficking/diffusion) also depend on the intracellular Ca^2+^ levels (Korvers et al., 2016; Färber and Kettenmann, 2006). This mechanism also partly explains the activated microglial phenotype observed after S-023-0515 treatment. Whether extracellular Ca^2+^ is entering the cells or is being released from the stores in the presence of S-023-0515 still remains to be determined. These scenarios can be tested by using a Ca^2+^ indicator such as fluo-4 in solutions with extracellular solutions containing 0 Ca^2+^ or by using Ca2 + store-depleting agents, including thapsigargin.

The findings of this work define the specific molecular components of the microglial inflammatory cascade that are dependent on the Hv_1_ channel function. The implementation of two different pharmacological tools enabled us to describe the specific effect of Hv_1_ channel activation and inhibition on microglial activation *in vitro*. While most of *in vivo* knock out (KO) studies demonstrated the effects of Hv_1_ channel deletion, they do not accurately reflect the effects of reduced gene expression. Some limitations of gene KO models include off-target effects such as alterations in signalling pathways, developmental compensations, and phenotype differences in specific cellular KO vs. whole animal KO can occur. For instance, Hv_1_ KO mice showed impaired Akt signalling pathway, while little is known regarding Hv_1_’s role in this signalling pathway (Capasso et al., 2010). Therefore, our study hugely complements the existing data from *in vivo* Hv_1_ KO models and also reveals a new NOX2-independent role of Hv_1_ channels in microglial cells.

### 3.1 Therapeutic potential of Hv_1_ channels

Pharmacological inhibition of Hv_1_ channels is clearly emerging as a viable strategy to alleviate neuroinflammation for the treatment for neurodegenerative disorders or chronic pain. Apart from the beneficial anti-neuroinflammatory effects as evident from the KO mice models, Hv_1_ channels also offers some potential therapeutic advantages. Firstly, Hv_1_ KO mice did not display any spontaneous pathologies, suggesting that its blockade will also not have any severe consequences on normal physiological functions (Sasaki et al., 2013). Secondly, targeting Hv_1_ channels is more valuable than direct inhibition of NOX2. Either deficiency or blockade of NOX2 completely abolishes ROS production that is normally advantageous and essential for supporting the functioning and viability of cells. Conversely, Hv_1_-deficient mice had a residual ROS release of approximately 30%, enough to support vital cellular functions such as innate immune responses (Ramsey et al., 2009). Lastly, since Hv_1_ channels are typically localized on the membrane surfaces, they can be easily accessed by small molecule modulators. On the other hand, Hv_1_ channel activation is beneficial for treating some conditions such as male infertility. In sperm cells, Hv_1_ channels are located on the principal piece (Miller et al., 2018) and mediate their hypermotility, capacitation and acrosome reaction. Activation of these channels with albumin has been previously shown to augment oocyte fertilization, and thus, Hv_1_ channel activators including S-023-0515 will also have a salutary effect depending on the disease condition.

### 3.2 Limitations of the current study

BV2 cells are being extensively used as an alternative model for primary microglia to study *in vitro* microglial functions due to their ease of use and reproducibility. Although BV2 cells display all the fundamental aspects of microglia, such as phagocytosis, proinflammatory cytokine production, NO and ROS generation (Henn et al., 2009; Das et al., 2015), they do not recapitulate all the nuances of *in vivo* microglial functions. Some of the major limitations of using BV2 cells include (i) A lower reactivity to LPS compared to the primary microglia (Das et al., 2016), (ii) Proteomic dissimilarities in the resting and activated states (Luan et al., 2022), (iii) Differences in TGFβ signalling and chemotactic capability (He et al., 2018), and (iv) Absence of interactions with other brain cell types. To increase the pathophysiological significance of our outcomes, the relevant results should be validated in the primary microglial cultures. Considering these functional and transcriptional differences, caution should be exercised while associating our BV2 cell line findings with microglial cells in the brain *in vivo* during inflammatory conditions.

## 4 Materials and methods

### 4.1 Cell culture

BV2 microglia cell cultures were cultured in DMEM/F-12 containing 10% heat-inactivated fetal bovine serum plus 1% penicillin/streptomycin and kept in a humidified incubator at 37°C, 5% CO_2_. Cells between passage 5–20, were plated at a density of 1–2 × 10^5^/ml for all experiments. Prior to experimentation, cells were seeded into either 12- or 96-well plates and allowed to adhere for 12 h. For electrophysiology experiments, cells were plated on 12 mm poly-lysine-treated glass coverslips. For RT-PCR, immunoblotting and immunostaining analysis, ROS, and phagocytosis assays, cells were pretreated with either 10 μM YHV98-4 or 10 μM S-023-0515 for 1 h and then treated with 100 ng/ml LPS for 24 h.

### 4.2 Knockdown of Hv_1_

BV2 cells at a density of 5 × 10^5^ in a 6-well plate were incubated for 18–20 h. Later, the medium was removed from the wells and replaced with fresh media. 10 μl of Lentiviral shRNA particles (mouse HVCN1; Origene-TL504897V and scramble; Origene–TR30021V) were added in culture medium with polybrene (final concentration 8 μg/ml) to the total volume of 1 ml DMEM/F-12 media and left for 72 h. Finally, the transduction efficiency was evaluated under the fluorescent microscope and gene knockdown was validated using immunocytochemistry, qPCR, western blot and patch clamp electrophysiology techniques.

### 4.3 Primary fluorescent-based screening assay

BV2 cells at a density of 2×10^5^ cells/well are seeded in a black-walled, clear-bottom 96-well microplate and were loaded with 1 μM BCECF-AM dye (Molecular Probes) for 25 min in dark at room temperature in standard buffer solution (SBS): 160 NaCl, 4.5 KCl, 2 CaCl2, 1 MgCl2, 5 HEPES; pH 7.4 and washed 3 times with SBS to remove the extracellular dye. NH_4_^+^ prepulse technique, as described earlier (Chen et al., 2020), was used to induce acid load in the cells. Briefly, the cells were covered with 30 mM NH4Cl solution for 5 min to induce acute acid load. This step initially alkalinizes the cell, and after approximately 2–3 min, the pH returns to near resting pH_i_. Subsequently, the extracellular NH4Cl is replaced with a Na^+^ free buffer: 135 mM N-Methyl glucamine, 5 mM KCl, 1.8 mM CaCl_2_, 1 mM MgCl2, 5.5 mM Glucose and 10 mM HEPES. The rapid removal of extracellular NH4Cl shifts the intracellular equilibrium towards acid production, resulting in a large acid load in the cell (~pH_i_ = 6.0). Once the cells are acidified, they are stimulated with a high K^+^-containing buffer (145 mm KCl, 5 mm glucose, 1 mm CaCl_2_, 1 mm MgCl_2_, 20 mm Tris, pH 8.0), which induces membrane depolarization and activates Hv_1_ channels. The high extracellular pH promotes the Hv_1_ channel opening, and the lack of external Na^+^ in this solution minimises the activity of the Na^+^/H^+^ exchanger (NHE1). Under these conditions, the total pH_i_ recovery is solely due to the activation of Hv_1_ channels. The intracellular pH changes in these acid-loaded cells were detected at an excitation wavelength of 488 nm and an emission wavelength of 535 nm using Flexstation 3 multi-mode plate reader (molecular devices).

### 4.4 Chemicals

YHV98-4 and its derivatives were synthesized in house. Reagents and starting materials for chemical synthesis were purchased from Aldrich, Alfa Aesar, TCI, Spectrochem and other commercial sources and used without further purification unless otherwise noted. The structures of the compounds were confirmed by H NMR and high-resolution mass spectra and its purity is determined using HPLC analysis. The synthesis routes and characterization of all the compounds are outlined in the Supplementary section. Salts for patch clamp electrophysiology and LPS from *E. coli* O111: B4 (L3012) were obtained from Sigma-Aldrich. 2′,7′-bis(2-carboxyethyl)-5(6)-carboxyfluorescein-acetoxymethyl ester (BCECF-AM) was purchased from Calbiochem. Compound solutions were prepared on the day of an experiment in 100% DMSO and used only for ~6 h. YHV98-4 and S-023-0515 stock solutions were kept at 50 mM concentration in DMSO. The desired concentrations of compounds were obtained by appropriate dilution in the external solution. DMSO was present at 0.02% (v/v) in the concentration of compound typically used in this study (10 μM) and at 0.2% (v/v) in the highest dose tested (100 μM).

### 4.5 Patch clamp electrophysiology

Whole-cell current recordings were performed on either BV2 cells that endogenously express Hv_1_ channels. Patch pipettes are fabricated from thick-wall borosilicate glass (1.5 mm O. D. x 0.86 mm I. D.) using a Sutter P-1000 puller and fire-polished. The obtained pipettes will have a resistance of 1.5–6 mΩ when filled with recording solutions. The intracellular solution contained: 75 mM NMDG, 180 mM MES, 15 mM Glucose, 3 mM MgCl_2_, 1 mM EGTA; pH adjusted to 6.4 with CsOH. The extracellular solution contained 75 mM NMDG, 180 mM HEPES, 15 mM Glucose, 3 mM MgCl_2_, and 1 mM EGTA; pH adjusted to 7.4 with CsOH. The osmolarity of these solutions was adjusted to 290–300 mm. No liquid junction potential correction was applied and all experiments were performed at room temperature (20–25°C). Currents were recorded using the Multiclamp 700A amplifier (Axon Instruments, Molecular Devices) with a low–pass filtering at 2 kHz, and digitized with the Axon Digidata 1440A at 1 ms. Clampex and Clampfit software (pClamp10; Axon Instruments) were used for stimulus generation and data acquisition.

### 4.6 Quantitative reverse transcription polymerase chain reaction (qRT-PCR)

Total RNA was isolated from BV2 cells using TRIZOL reagent (Invitrogen Co, Grand Island, NT, USA) according to the manufacturer’s instructions. cDNA was synthesized from 1 μg of total RNA by the High-Capacity cDNA Reverse Transcription Kits (Applied Biosystems) according to the manufacturer’s protocol. Subsequently, RT-PCR was conducted using CFX96 Touch Real-Time PCR Detection System (BIO-RAD, USA) with a SYBR Green Master Mix (Applied Biosystems (Excel Taq™ 2X Fast Q-PCR Master Mix, SMOBIO, Hsinchu, Taiwan) using the following primers: iNOS sense: 5′-CAACAGGGAGAAAGCGCAAA-3′, antisense: 5′-CAGGTCACTTTGGTAGGATTT-3′; IL-6 sense: 5′-GAGGATACCACTCCCAACAGACC-3′, antisense: 5′-AAGTGCATCATCGTTGTTCATACA-3′; TNF-α sense: 5′-AGGGATGAGAAGTTCCCAAATG-3′, antisense: 5′-TGTGAGGGTCTGGG CCATA-3′; β-actin sense: 5′-GACCTCTATGCCAACACAGT-3′, antisense: 5′-AGTACTTGCGCTCAGGAGGA-3′. The mRNA expression levels of iNOS, IL-6, and TNF-α were normalised to actin. Results are reported as fold-change in gene expression, determined using the delta–delta Ct (ΔΔ^Ct^) method using the threshold cycle (Ct) value for actin and the respective gene of interest in each sample.

### 4.7 Immunocytochemistry

BV2 cells were seeded in 24 well plates containing with poly-l-lysine treated 12 mm cover slips at a density of 2.5 × 10^5^ cells/well and maintained overnight. Cells were fixed in ice cold 2% paraformaldehyde solution (prepared in PBS) and incubated for 15 min at 4°C. 0.5% Triton X-100 (prepared in PBS, pH 7.2) was used for permeabilization and cells were incubated for 30 min at room temperature. Blocking buffer (3% Bovine serum albumin, 3% horse serum, 0.3% Triton X-100, 0.3% Sodium Azide) was used for blocking and the cells were incubated with primary antibody anti-HVCN1 (1:100; Alomone labs), anti-Iba1 (1:250; Novus Biologicals) anti-Phospho NFKB p65 (1:100; Bioss) and overnight at 4°C. Next, the cells were washed with PBS and incubated with secondary antibody (Alexa fluor-488, 1:1000, AbClonal) for 1 h in dark. DAPI (incubated along with secondary antibody) was used as a nuclear counterstain. After washing the cells, cover slips were mounted on the glass slides using mounting media (DPX). Images were acquired on confocal microscope.

### 4.8 Nitrite assay

The Griess reagent assay (ThermoFisher #G7921, USA) was used to quantify media nitrite levels in BV2 cells. Cells (5 × 10^5^/well) were seeded in 96-well plates and pretreated with the indicated concentrations of compounds for 24 h. Next, the conditioned media were added to the equal volume of Griess reagent, and the nitrite levels were evaluated by measuring the absorbance at the 542 nm wavelength. A standard curve was prepared from nitrite-containing samples and used to determine sample concentrations.

### 4.9 Flow cytometry

Cell surface expression of CD16/32 (M1 marker), CD206 (M2 marker) in response to different treatment groups was analyzed by Flow cytometry. In brief, cells were allowed to grow up to 70–80% confluence and then harvested with Accutase (Invitrogen) for single-cell suspension in FACS buffer (PBS with 0.1% BSA). Cells were stained with fluorochrome-conjugated antibodies in FACS buffer for 30–45 min at room temperature in the dark. After washing and centrifugation, cell pellets were resuspended in FACS buffer and analyzed by FACS Lyric (BD). Acquired data were analyzed using and data analysis was performed using Floreada.io software.^1^ Results were expressed as mean % positive cells.

### 4.10 Western blotting

BV2 cells were homogenized using RIPA Lysis Buffer supplemented with protease inhibitors. Protein concentration was measured by BCA and the cell extracts (30 μg) were loaded onto SDS-polyacrylamide gels and transferred onto a PVDF membrane (Merck Millipore). After blocking with 5% BSA at room temperature for 1 h, membranes were incubated at 4°C overnight with primary antibodies against Hvcn 1 (1:200, Alomone Labs), p65 (1:200, Cell Signaling Technology, USA), p-p65 (1:200, Cell Signaling Technology, USA), β-actin (1:1000, Cell Signaling Technology, USA). The following day, membranes were incubated with HRP-conjugated secondary antibodies (1,5,000, Affinity bioscience; USA) for 2 h. Immunoblots were developed with an enhanced chemiluminescence system (CLARITY™ Western ECL Substrate, Bio-Rad, USA) using Amersham ImageQuant 800, and densitometric analysis was performed by ImageJ software.

### 4.11 Cell viability assay

Cell viability of BV2 cells exposed to different concentrations of test compounds was evaluated using MTT assay. Briefly, 1× 10^5^ cells were seeded in 96 well-plates and after 24 h the cells were treated with increasing concentrations of YHV98-4 and S-023-0515 (from 0.1 μM to 100 μM) in serum-free medium, without phenol red and incubated for 24 h. Afterwards, 3-(4,5-dimethylthiazol-2-yl)-2,5-diphenyltetrazolium bromide (MTT) solution was added to each well and incubated for 4 h. Finally, 100 μl solution was added to dissolve formazan and absorbance was measured at 570 nm, using 620 nm as wavelength reference.

### 4.12 Reactive oxygen species quantification

The fluorescent dye 2′,7’-Dichlorofluorescin-diacetate H2DCFDA (Millipore, catalog number: 287810) and mitoSOX red (Invitrogen, catalog number: M36008) was used to determine intracellular and mitochondrial reactive oxygen species (ROS), respectively. WT BV2 microglial cells were seeded into black-walled, clear-bottom 96-well plates and then stimulated with LPS, LPS + YHV98-4, S-023-0515, and LPS + S-023-0515 for 24 h. Next, the cells were incubated with either 10 μM H2DCFDA or 5 μM MitoSOX red for 20 min at 37°C in the dark and then washed thrice with HBSS media to remove any residual dye. Later images were acquired on a fluorescent microscope (DMI-6000, Leica) using GFP filter for H2DCFDA and TRITC filter for mitoSOX red. Simultaneously, fluorescent intensity was measured for H2DCFDA on a Flexstation 3 multi-mode plate reader at excitation and emission wavelengths of 490 nm and 520 nm, respectively. The fluorescent values for three wells were averaged for each plate.

### 4.13 Phagocytosis assay

BV2 microglia were plated at a density of 2 × 10^5^ cells/well on 1.5-mm (Muzio et al., 2021) coverslips and then treated with LPS, LPS + YHV98-4, S-023-0515, and LPS + S-023-0515 for 24 h. Then, fluorescence-labelled latex beads of 1 μM diameter (Sigma-Aldrich, catalog number: L3030) were added at a concentration of 5 μl/ml for 2 h at 37°C. The cells were washed three times with PBS to remove the non-phagocytized beads and fixed with 4% paraformaldehyde. Next, phagocytosis of the beads by microglia was observed under a fluorescence-inverted microscope. Phagocytosis quantification was done by counting the number of phagocytosed beads in BV2 microglial cells over total number of cells in at least 4 to 6 microscopic fields using ImageJ software.

### 4.14 *In silico* activity prediction

To predict the potential biological activities and molecular targets of YHV98-4 and S-023-0515, the Prediction of Activity Spectra for Substances (PASS) online server was utilised^2^ (Pogodin et al., 2015). The chemical structures of these two compounds were submitted in SMILES format. PASS predicts probable biological activities based on the structure–activity relationship derived from a large training dataset of biologically active compounds from CHEMBEL. The results are expressed in terms of a confidence score, which gives the probability of being active for a particular target. Confidence is the difference between probabilities for a chemical compound to interact and not to interact with a particular target. The higher confidence means a higher chance of the positive prediction being true. The targets with scores greater than 0.7 are considered potentially significant, which indicates a high likelihood that the compound exhibits the predicted activity under physiological conditions.

### 4.15 Statistical analysis

Data are expressed as mean ± SEM and were analyzed by unpaired Student t-test or by Ordinary one-way ANOVA, using the Dunnett’s or Tukey’s multiple comparisons test as appropriate to determine statistical significance (*p < 0.05, **p < 0.01, and ***p < 0.001 vs. Control and (#p < 0.05, ##p < 0.01, and ### p < 0.001 vs. LPS). In the data presented, N and n indicate independent experiments and technical replicates, respectively.

## Data Availability

The original contributions presented in the study are included in the article/Supplementary material, further inquiries can be directed to the corresponding author.

## Footnotes

^1^ https://floreada.io/analysis

^2^ http://www.pharmaexpert.ru/passonline/
