# Supplementary material for: Validation of Hv1 channel functions in BV2 microglial cells using small molecule modulators
Source: Front Cell Neurosci. 2025 Jul 29;19:1624224. doi: 10.3389/fncel.2025.1624224 (PMC12340723; doi:10.3389/fncel.2025.1624224)
Supplement: Supplementary file 1 [file Data_Sheet_1.docx]

**Validation of Hv_1_ channel functions in BV2 microglial cells using small molecule modulators.**

Ashutosh Sharma^1,3^, Nandini kale^3^, Priyanka Yadav^1,3^, Shivani Yadav^1,3^, Madhavi Ranawat^1,3,^

Valmik Shinde^2,3^ and Aravind Singh Kshatri^1,3^

^1^Neuroscience and Ageing Biology Division, CSIR- Central Drug Research Institute (CDRI), Lucknow, 226031, India

^2^Medicinal and Process Chemistry Division, CSIR- CDRI, Lucknow, 226031, India

^3^Academy of Scientific and Innovative Research, Ghaziabad, 201002, India.

*To whom correspondence should be addressed:

Dr. Aravind Kshatri

Department of Neuroscience & Ageing Biology,

Central Drug Research Institute,

Lucknow - 226031, India

Tel: 91-522-2772450 (Extn-4545/46)

E-mail: aravind.kshatri@cdri.res.in

Keywords:

Microglía, Hv_1_ channels, neuroinflammation, activator, inhibitor, signalling pathway

**Supplementary information**

**Supplemental Tables**

**Table 1: List of predicted direct targets for YHV98-4**

| **S.No** | **Target name** | **Confidence score** |
| --- | --- | --- |
| 1 | Tyrosyl-DNA phosphodiesterase 1 | 0.51 |
| 2 | Homeodomain-interacting protein kinase 3 | 0.50 |
| 3 | Serine/threonine-protein kinase Nek11 | 0.48 |
| 4 | Dual specificity mitogen-activated protein kinase kinase 7 | 0.41 |
| 5 | Homeodomain-interacting protein kinase 2 | 0.41 |
| 6 | Serine/threonine-protein kinase PRKX | 0.40 |
| 7 | Casein kinase I delta | 0.39 |
| 8 | Polyadenylate-binding protein 1 | 0.38 |
| 9 | Serine/threonine-protein kinase WNK3 | 0.37 |
| 10 | PAS domain-containing serine/threonine-protein kinase | 0.37 |
| 11 | MAP kinase p38 delta | 0.37 |
| 12 | MAP kinase-activated protein kinase 5 | 0.35 |
| 13 | Casein kinase II alpha (prime) | 0.35 |
| 14 | Eukaryotic translation initiation factor 4H | 0.35 |
| 15 | Muscle, skeletal receptor tyrosine protein kinase | 0.32 |

**Table 2. List of predicted direct targets for S-023-0515**

| **S.No** | **Target name** | **Confidence score** |
| --- | --- | --- |
| 1 | Serine/threonine-protein kinase Nek11 | 0.50 |
| 2 | Tyrosyl-DNA phosphodiesterase 1 | 0.49 |
| 3 | Homeodomain-interacting protein kinase 3 | 0.47 |
| 4 | Dual specificity mitogen-activated protein kinase kinase 7 | 0.44 |
| 5 | Ephrin type-B receptor 3 | 0.41 |
| 6 | Polyadenylate-binding protein 1 | 0.40 |
| 7 | Eukaryotic translation initiation factor 4H | 0.40 |
| 8 | Homeodomain-interacting protein kinase 2 | 0.39 |
| 9 | Serine/threonine-protein kinase TAO2 | 0.37 |
| 10 | Muscle, skeletal receptor tyrosine protein kinase | 0.36 |
| 11 | Casein kinase I delta | 0.36 |
| 12 | Serine/threonine-protein kinase PRKX | 0.35 |
| 13 | MAP kinase p38 delta | 0.34 |
| 14 | Casein kinase I isoform gamma-3 | 0.33 |
| 15 | Macrophage-stimulating protein receptor | 0.32 |

**Supplemental Figures**

**Figure S1: In-house synthesis of a library of compounds belonging to YHV98-4 family.**


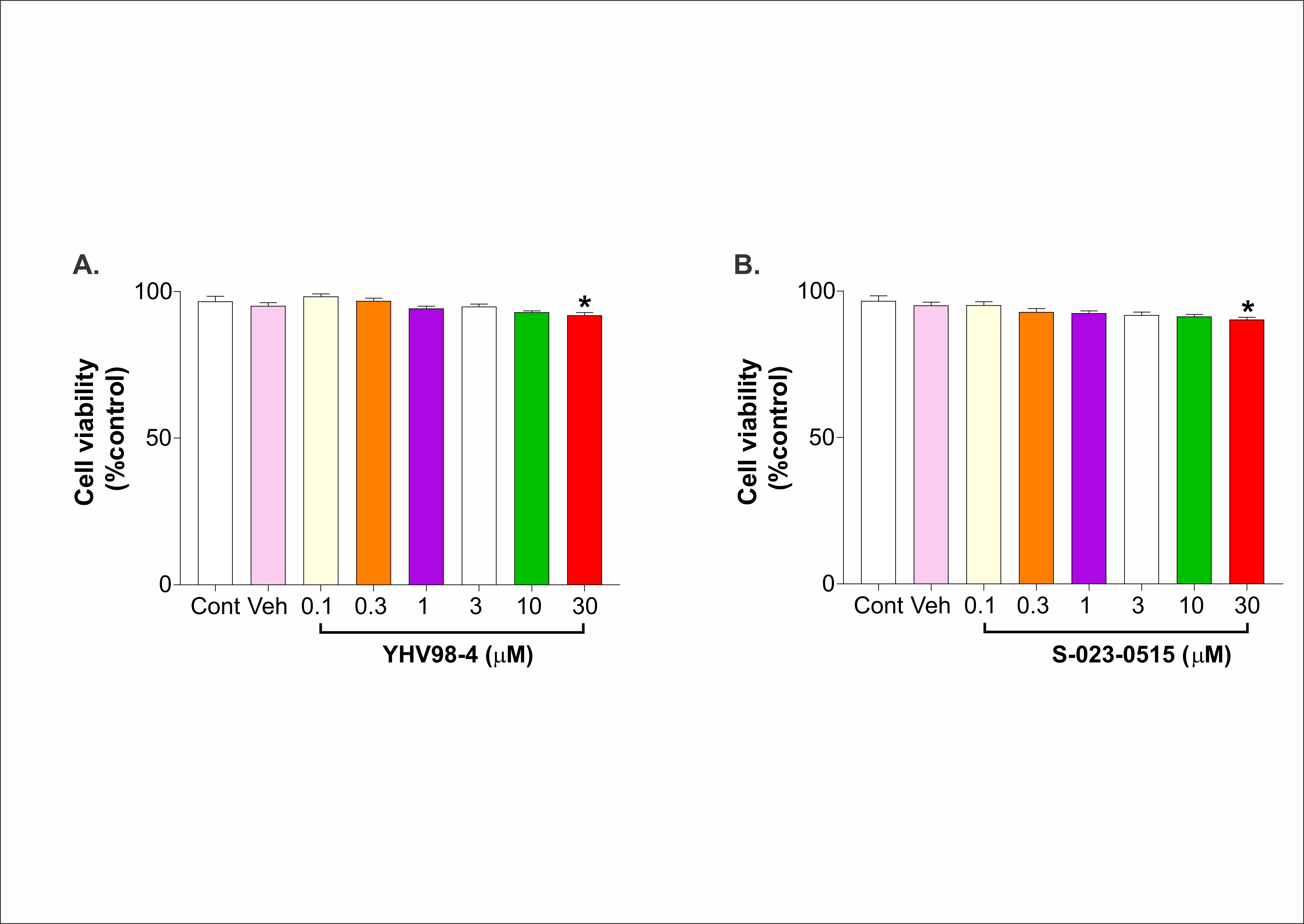


**Figure S2: BV2 cell viability is largely unaffected by YHV98-4 and S-023-0515 treatment.** (A, B) MTT assay was performed after treating the cells with different concentrations of YHV98-4 (A) and S-023-0515 (B) for 24 h. 0.1% DMSO was used as a vehicle. All the data are expressed as means ± SEM and were analysed using ordinary one-way ANOVA followed by Tukey’s post hoc test. N=3 independent experiments. *p < 0.05 compared to control.

**Supplemental methods**

**Chemical Synthesis and Characterization Data:**

Reagents and starting materials were purchased from Aldrich, Alfa Aesar, TCI, Spectrochem and other commercial sources and used without further purification unless otherwise noted. All the reactions were performed using borosil sealed tube vial with dry solvents under inert (nitrogen) atmosphere. Anhydrous tetrahydrofuran (THF), Dimethyl formamide (DMF) were used were purchased from Merck and Thermofisher. The tert-butyl methyl ether (TBME) ethyl acetate (EtOAc), and dichloromethane (DCM) were purchased commercially and were used without further purification, unless otherwise stated. Column chromatography was done in 60Å-120Å silica gel of Merck company. All spectra ^1^H NMR, ^13^C NMR, recorded on Bruker AV 400 MHz spectrometer in CDCl_3_ using TMS as internal standards. The following abbreviations were used to describe peak splitting patterns when appropriate: s = singlet, d = doublet, t = triplet, q = quartet, dd = doublet of doublet, m = multiplet. The coupling constants, J, were reported in Hertz (Hz). IR, ESI-MS, HRMS analysis was performed using Q-TOF mass spectrometer of the SAIF Division in CSIR-CDRI Lucknow.

**General procedure A: Synthesis of (E)-4-oxo-4-(phenylamino)but-2-enoic acid.**

To a solution of substituted amines (1.0 eq.) in tetrahydrofuran was added maleic anhydride (1.2 eq.) in tetrahydrofuran at room temperature. The resulting mixture was stirred at room temperature overnight. Upon the completion of the reaction (TLC monitoring), the thick white precipitate was filtered off, washed with tetrahydrofuran, and dried to obtain the substituted (E)-4-oxo-(phenylamino)but-2-enoic acid.

**General procedure B: Synthesis of 2-(3-oxo-3,4-dihydro-2H-benzo[b][1,4]thiazin-2-yl)-N-phenylacetamide.**

2-amino thiophenol (1.09 eq.) was added to a solution of compound **3** (1.0 eq.) in DMF at room temperature and the mixture was then heated to 150 ^o^C for 5-6 hours. Upon the completion of reaction (TLC monitoring) reaction mixture was cooled to room temperature and poured into water. The resulting solid was filtered off to obtain the crude product. The crude product was washed with tert-butyl methyl ether/ tetrahydrofuran (4:1) or with diethyl ether and filtered to obtain the desired product.

**YHV98-4: N-(4-chlorophenyl)-2-(3-oxo-3,4-dihydro-2H-benzo[b][1,4]thiazin-2-yl)acetamide**

The title compound was prepared according to general procedure, and isolated as off-white solid in 88% yield; Rf = 0.5 in EtOAc: Hex (1:1); mp: 275 ^o^C.

**^1^H NMR** (501 MHz, DMSO-*d6*) δ 10.67 (bs, 1H), 10.17 (s, 1H), 7.60 (d, *J* = 8.5 Hz, 2H), 7.35 (dd, *J* = 8.8, 1.6 Hz, 3H), 7.25 – 7.17 (m, 1H), 7.05 – 6.97 (m, 2H), 3.94 (dd, *J* = 8.5, 5.7 Hz, 1H), 3.04 – 2.93 (m, 1H), 2.59 (dd, *J* = 15.5, 8.6 Hz, 1H).

**^13^C NMR** (126 MHz, DMSO) δ 168.04, 166.40, 138.39, 137.36, 129.12, 128.22, 127.70, 127.33, 123.63, 121.08, 118.50, 117.59, 38.16, 36.62.

**IR (CH_2_Cl_2_) *ν*** 3279.95, 3199.58, 2910.92, 1663.02, 1394.57, 749.60 cm^-1^

**Mol wt** 332.80; **ES-MS^+^**: 333.2

**HPLC purity:**  The compound was found to be 97 % pure at 220 nm on analytical RP-HPLC.

**S-023-0515: N-(2-chlorophenyl)-2-(3-oxo-3,4-dihydro-2H-benzo[b][1,4]thiazin-2-yl)acetamide**

This compound was prepared according to the general procedure, and isolated as white solid in 64% yield; Rf = 0.5 in EtOAc: Hex (1:1); mp: 228 ^o^C;

**^1^H NMR** (400 MHz, DMSO- *d6*) δ 10.69 (bs, 1H), 9.66 (s, 1H), 7.76 – 7.70 (m, 1H), 7.49 (dd, *J* = 8.0, 1.5 Hz, 1H), 7.36 – 7.30 (m, 2H), 7.25 – 7.16 (m, 2H), 7.01 (dd, *J* = 8.0, 6.9 Hz, 2H), 3.94 (dd, *J* = 8.6, 5.6 Hz, 1H), 3.03 (dd, *J* = 15.5, 5.7 Hz, 1H), 2.72 (dd, *J* = 15.7, 8.4 Hz, 1H).

**^13^C NMR** (101 MHz, DMSO- *d6*) δ 167.95, 165.98, 136.88, 134.73, 129.44, 127.18, 126.25, 126.02, 123.12, 118.08, 117.08, 37.81, 35.57.

**IR (CH_2_Cl_2_) *ν*** 3279.95, 3199.58, 2910.92, 1663.02, 1394.57, 749.60 cm^-1^

**Mol wt:** 332.80; **ES-MS^+^**: 333.3

**HPLC purity:**  The compound was found to be 96 % pure at 220 nm on analytical RP-HPLC.

**S-023-0518:N-((3R,5S)-adamantan-1-yl)-2-(3-oxo-3,4-dihydro-2H-benzo[b][1,4]thiazin-2-yl)acetamide**

This compound was prepared according to the general procedure, and isolated as brown solid; 49.62% in yield; Rf = 0.5 in EtOAc: Hex (1:1); mp: 152.2 ^o^C;

**^1^H NMR** (400 MHz DMSO- *d6*) δ 8.53 (bs, 1H), 7.21 (dd, *J* = 7.8, 1.4 Hz, 1H), 7.06 (dd, *J* = 7.7, 1.5 Hz, 1H), 6.92 (dd, *J* = 7.7, 1.3 Hz, 1H), 6.77 (dd, *J* = 8.0, 1.3 Hz, 1H), 5.44 (s, 1H), 3.87 (d, *J* = 6.8 Hz, 1H), 2.72 (dd, *J* = 14.7, 6.7 Hz, 1H), 2.29 (dd, *J* = 14.7, 7.0 Hz, 1H), 1.92 (dd, *J* = 23.7, 3.0 Hz, 11H), 1.19 – 1.10 (m, 6H).

**^13^C NMR** (101 MHz, DMSO- *d6*) δ 168.05, 167.87, 136.07, 128.21, 127.45, 124.14, 120.09, 117.26, 52.44, 41.69, 38.68, 37.01, 36.46, 29.56.

**IR (CH_2_Cl_2_) *ν*** 3331.71, 2916.45, 2858.70, 1666.93, 1382.42, 1035.28 cm^-1^

**Mol wt:** 356.48; **ES-MS^+^**: 357.4.

**HPLC purity:**  The compound was found to be 96 % pure at 220 nm on analytical RP-HPLC.

**S-023-0520: 2-(3-oxo-3,4-dihydro-2H-benzo[b][1,4]thiazin-2-yl)-N-(p-tolyl)acetamide**

This compound was prepared according to the general procedure, and isolated as grey solid in 30% yield; 0 Rf = 0.5 in EtOAc: Hex (1:1); mp: 252^o^C;

**^1^H NMR** (400 MHz, DMSO- *d6*) δ 10.67 (bs, 1H), 9.95 (s, 1H), 7.48 – 7.43 (m, 2H), 7.33 (dd, *J* = 7.7, 1.5 Hz, 1H), 7.25 – 7.18 (m, 1H), 7.12 – 7.08 (m, 2H), 7.04 – 6.97 (m, 2H), 3.93 (dd, *J* = 8.8, 5.5 Hz, 1H), 2.95 (dd, *J* = 15.5, 5.5 Hz, 1H), 2.60 – 2.52 (m, 1H), 2.24 (s, 3H).

**^13^C NMR** (101 MHz, DMSO- *d6*) δ 167.55, 166.48, 137.35, 136.97, 132.65, 129.56, 128.23, 127.68, 123.62, 119.52, 118.51, 117.55, 38.26, 36.54, 20.91.

**Mol wt:** 312.39; **ES-MS^+^**: 313.1.

**HPLC purity:**  The compound was found to be 93 % pure at 220 nm on analytical RP-HPLC.

**S-024-0755: N-(2-aminophenyl)-2-(3-oxo-3, 4-dihydro-2H-benzo[b][1,4]thiazin-2-yl)acetamide**

This compound was prepared according to the general procedure and isolated as off white solid in 49.62% yield; Rf = 0.4 in MeOH: DCM (1:9); mp: 259.2 ^o^C;

**^1^H NMR** (400 MHz, DMSO- *d6*) δ 10.69 (bs, 1H), 9.42 (s, 1H), 7.39 (dd, *J* = 7.9, 1.4 Hz, 1H), 7.34 (dd, *J* = 7.7, 1.4 Hz, 1H), 7.25 – 7.18 (m, 2H), 7.15 (td, *J* = 7.6, 1.8 Hz, 1H), 7.08 (td, *J* = 7.4, 1.5 Hz, 1H), 7.05 – 6.98 (m, 2H), 3.94 (dd, *J* = 8.8, 5.6 Hz, 1H), 2.98 (dd, *J* = 15.2, 5.6 Hz, 1H), 2.64 (dd, *J* = 15.2, 8.9 Hz, 1H), 2.21 (s, 3H).

**^13^C NMR** (101 MHz, DMSO- *d6*) δ 167.39, 166.07, 136.89, 136.10, 131.71, 130.26, 127.72, 127.19, 125.86, 125.04, 123.14, 118.02, 117.07, 38.04, 35.62, 17.85.

**IR (CH_2_Cl_2_) *ν*** 3421.08, 3271.95, 3200.71, 1666.31, 1538.13, 1264.57 cm^-1^

**Mol wt:** 312.39; **ES-MS^+^**: 313.1.

**HPLC purity:**  The compound was found to be 92 % pure at 220 nm on analytical RP-HPLC.

**S-024-0756: N-(2-methoxyphenyl)-2-(3-oxo-3,4-dihydro-2H-benzo[b][1,4]thiazin-2-yl)acetamide**

This compound was prepared according to the general procedure, and isolated as pale yellow solid in 26.08% yield; Rf = 0.5 in MeOH: DCM (1:9); mp: 183.6 ^o^C;

**^1^H NMR** (400 MHz, DMSO- *d6*) δ 10.67 (bs, 1H), 9.31 (s, 1H), 7.99 (dd, *J* = 7.9, 1.6 Hz, 1H), 7.33 (dd, *J* = 7.7, 1.4 Hz, 1H), 7.21 (d, *J* = 1.0 Hz, 1H), 7.11 – 6.96 (m, 5H), 6.93 – 6.86 (m, 1H), 3.98 – 3.90 (m, 1H), 3.81 (s, 3H), 3.01 (dd, *J* = 15.6, 5.6 Hz, 1H), 2.81 – 2.70 (m, 1H).

**^13^C NMR** (101 MHz, DMSO- *d6*) δ 167.65, 166.13, 149.37, 136.90, 127.68, 127.15, 124.35, 123.09, 121.71, 120.20, 118.20, 117.06, 111.18, 55.64, 37.84, 35.89.

**IR (CH_2_Cl_2_) *ν*** 3251.61, 3117.03, 2970.11, 1776.31, 1659.57, 747.94 cm^-1^

**Mol wt:** 328.39; **ES-MS^+^**: 329.1.

**HPLC purity:**  The compound was found to be 97 % pure at 220 nm on analytical RP-HPLC.

**S-024-0752: N-(2-bromophenyl)-2-(3-oxo-3,4-dihydro-2H-benzo[b][1,4]thiazin-2-yl)acetamide**

This compound was prepared according to the general procedure, and isolated as grey solid in 52.4% yield; Rf = 0.5 in MeOH: DCM (1:9); mp: 239.8 ^o^C;

**^1^H NMR** (400 MHz, DMSO-*d6*) δ 10.69 (bs, 1H), 9.61 (s, 1H), 7.63 (ddd, *J* = 15.4, 8.4, 1.5 Hz, 2H), 7.40 – 7.33 (m, 2H), 7.22 (ddd, *J* = 8.6, 7.3, 1.5 Hz, 1H), 7.13 (td, *J* = 7.7, 1.6 Hz, 1H), 7.04 – 6.98 (m, 2H), 3.94 (dd, *J* = 8.7, 5.5 Hz, 1H), 3.01 (dd, *J* = 15.4, 5.6 Hz, 1H), 2.74 – 2.64 (m, 1H).

**^13^C NMR** (126 MHz, DMSO- *d6*) δ 168.32, 166.46, 137.35, 136.54, 133.14, 128.42, 128.24, 127.54, 123.64, 118.52, 117.57, 38.32, 35.99.

**IR (CH_2_Cl_2_) *ν*** 3780.19, 3270.24, 2311.86, 1778.76, 1659.93, 744.21 cm^-1^

**Mol wt:** 378.26; **ES-MS^+^**: 379.

**HPLC purity:**  The compound was found to be 98 % pure at 220 nm on analytical RP-HPLC.

**S-024-0754:N-(2-bromo-4-(trifluoromethoxy)phenyl)-2-(3-oxo-3,4-dihydro-2H-benzo[b][1,4]thiazin-2-yl)acetamide**

This compound was prepared according to the general procedure, and isolated as off-white solid in 54.47% yield; Rf = 0.5 in MeOH: DCM (1:9); mp: 243.6 ^o^C;

**^1^H NMR** (400 MHz, DMSO- *d6*) δ 10.70 (bs, 1H), 9.75 (s, 1H), 7.77 – 7.71 (m, 2H), 7.43 (ddd, *J* = 8.9, 2.8, 1.2 Hz, 1H), 7.34 (dd, *J* = 7.8, 1.4 Hz, 1H), 7.21 (ddd, *J* = 8.4, 7.3, 1.4 Hz, 1H), 7.06 – 6.97 (m, 2H), 3.95 (dd, *J* = 8.6, 5.7 Hz, 1H), 3.04 (dd, *J* = 15.5, 5.7 Hz, 1H), 2.71 (dd, *J* = 15.5, 8.7 Hz, 1H).

**^13^C NMR** (101 MHz, DMSO- *d6*) δ 168.12, 165.92, 145.19, 136.87, 135.65, 127.74, 127.21, 125.45, 123.80, 123.15, 121.25, 120.82, 118.69, 118.00, 117.10, 37.80, 35.50.

**IR (CH_2_Cl_2_) *ν*** 3449.22, 3194.78, 2363.46, 1783.97, 1669.69, 1167.47 cm^-1^

**Mol wt:** 461.25; **ES-MS^+^**: 461.0.

**HPLC purity:**  The compound was found to be 97 % pure at 220 nm on analytical RP-HPLC.

**S-024-0753: N-(2-iodophenyl)-2-(3-oxo-3,4-dihydro-2H-benzo[b][1,4]thiazin-2-yl)acetamide**

This compound was prepared according to the general procedure, and isolated as grey solid in 84.03% yield; Rf = 0.5 in MeOH: DCM (1:9); mp: 244.5 ^o^C;

**^1^H NMR** (400 MHz, DMSO- *d6*) δ 9.84 (bs, 1H), 8.72 (s, 1H), 7.02 (dd, *J* = 7.8, 1.4 Hz, 1H), 6.61 – 6.46 (m, 3H), 6.36 (td, *J* = 7.7, 1.5 Hz, 1H), 6.20 – 6.10 (m, 3H), 3.09 (dd, *J* = 9.1, 5.2 Hz, 1H), 2.14 (dd, *J* = 15.5, 5.3 Hz, 1H), 1.78 (dd, *J* = 15.4, 9.1 Hz, 1H).

**^13^C NMR** (101 MHz, DMSO- *d6*) δ 167.66, 165.98, 139.33, 138.94, 136.87, 128.61, 127.84, 127.72, 127.20, 123.18, 117.99, 117.07, 37.90, 35.50.

**IR (CH_2_Cl_2_) *ν*** 3428.85, 3259.21, 3110.96, 2140.81, 1658.81, 746.60 cm^-1^

**Mol wt:** 424.26; **ES-MS^+^**: 425.0.

**HPLC purity:**  The compound was found to be 90 % pure at 220 nm on analytical RP-HPLC.

**S-024-0758: N-(2,4-dichlorophenyl)-2-(3-oxo-3,4-dihydro-2H-benzo[b][1,4]thiazin-2-yl)acetamide**

This compound was prepared according to the general procedure and isolated as grey solid in 43% yield; Rf = 0.4 in MeOH: DCM (1:9); mp: 237.2 ^o^C;

**^1^H NMR** (400 MHz, DMSO-*d6*) δ 10.70 (bs, 1H), 9.74 (s, 1H), 7.78 (d, *J* = 8.7 Hz, 1H), 7.64 (d, *J* = 2.4 Hz, 1H), 7.41 (dd, *J* = 8.8, 2.4 Hz, 1H), 7.33 (dd, *J* = 7.7, 1.5 Hz, 1H), 7.21 (ddd, *J* = 8.5, 7.4, 1.5 Hz, 1H), 7.08 – 6.96 (m, 2H), 3.94 (dd, *J* = 8.4, 5.8 Hz, 1H), 3.05 (dd, *J* = 15.5, 5.8 Hz, 1H), 2.73 (dd, *J* = 15.5, 8.5 Hz, 1H).

**^13^C NMR** (126 MHz, DMSO-*d6*) δ 168.16, 165.96, 136.88, 133.93, 128.90, 127.71, 127.50, 127.22, 123.15, 118.07, 117.12, 37.76, 35.58.

**IR (CH_2_Cl_2_) *ν*** 3924.41, 3294.25, 3073.38, 2291.34, 1659.77, 751.90 cm^-1^

**Mol wt:** 366.24; **ES-MS^+^**: 367.0.

**HPLC purity:**  The compound was found to be 100 % pure at 220 nm on analytical RP-HPLC.

**S-023-0519:2-(3-oxo-3,4-dihydro-2H-benzo[b][1,4]thiazin-2-yl)-N-(3-(trifluoromethyl)phenyl )acetamide**

This compound was prepared according to the general procedure, isolated as white solid in 31% yield; Rf = 0.4 in EtOAc: Hex (1:1); mp: 227^o^C;

**^1^H NMR** (400 MHz, DMSO- *d6*) δ 10.70 (bs, 1H), 10.40 (s, 1H), 8.09 (t, *J* = 2.0 Hz, 1H), 7.73 (d, *J* = 8.2 Hz, 1H), 7.55 (t, *J* = 8.0 Hz, 1H), 7.43 – 7.32 (m, 2H), 7.26 – 7.19 (m, 1H), 7.04 – 6.97 (m, 2H), 3.95 (dd, *J* = 8.4, 5.8 Hz, 1H), 3.00 (dd, *J* = 15.5, 5.9 Hz, 1H), 2.62 (dd, *J* = 15.5, 8.5 Hz, 1H).

**^13^C NMR** (126 MHz, DMSO- *d6*) δ 168.03, 166.49, 138.46, 135.79, 128.51, 126.89, 126.31, 122.71, 121.92, 119.20, 117.93, 116.19, 115.23, 37.34, 35.28.

**IR (CH_2_Cl_2_) *ν*** 3425.08, 3256.79, 2964.79, 1778.86, 1656.30, 1113.20 cm^-1^

**Mol wt:** 366.36; **ES-MS^+^**: 367.3.

**HPLC purity:**  The compound was found to be 100 % pure at 220 nm on analytical RP-HPLC.

**S-024-0757: 2-(3-oxo-3,4-dihydro-2H-benzo[b][1,4]thiazin-2-yl)-N-(1-phenylethyl)acetamide**

This compound was prepared according to the general procedure, and isolated as yellow solid in 57% yield; Rf = 0.6 in MeOH: DCM (1:9); mp: 235.4 ^o^C;

**^1^H NMR** (400 MHz, DMSO- *d6*) δ 10.64 (bs, 1H), 8.42 (dd, *J* = 8.0, 2.9 Hz, 1H), 7.41 – 7.16 (m, 7H), 7.04 – 6.94 (m, 2H), 3.83 (ddd, *J* = 10.5, 9.1, 5.2 Hz, 1H), 2.85 – 2.71 (m, 1H), 2.40 (ddd, *J* = 15.2, 9.2, 2.0 Hz, 1H), 1.33 (dd, *J* = 9.2, 7.0 Hz, 3H).

**^13^C NMR** (101 MHz, DMSO- *d6*) δ 167.47, 166.09, 144.40, 136.86, 128.17, 127.68, 127.12, 126.56, 125.93, 123.09, 118.17, 117.00, 47.94, 37.84, 35.11, 22.56.

**IR (CH_2_Cl_2_) *ν*** 3922.31, 3283.86, 3196.89, 2281.37, 1674.39, 1641.75 cm^-1^

**Mol wt:** 326.41; **ES-MS^+^**: 327.1.

**HPLC purity:**  The compound was found to be 94 % pure at 220 nm on analytical RP-HPLC.

**S-023-0523: N-benzhydryl-2-(3-oxo-3,4-dihydro-2H-benzo[b][1,4]thiazin-2-yl)acetamide**

This compound was prepared according to the general procedure, and isolated as grey solid in 36% yield; Rf = 0.5 in EtOAc: Hex (1:1); mp: 267 ^o^C;

**^1^H NMR** (400 MHz, DMSO- *d6*) δ 10.66 (bs, 1H), 8.90 (d, *J* = 8.6 Hz, 1H), 7.40 – 7.16 (m, 13H), 7.05 – 6.95 (m, 2H), 6.13 (d, *J* = 8.5 Hz, 1H), 3.87 (dd, *J* = 9.1, 5.3 Hz, 1H), 2.85 (dd, *J* = 15.2, 5.3 Hz, 1H), 2.54 (d, *J* = 9.1 Hz, 1H).

**^13^C NMR** (101 MHz, DMSO- *d6*) δ 167.55, 165.88, 128.09, 127.47, 127.15, 126.72, 122.90, 116.79, 55.85, 39.31, 37.82, 34.79.

**IR (CH_2_Cl_2_) *ν*** 3421.71, 3308.03, 2917.58, 2323.79, 1642.42, 1527.51 cm^-1^

**Mol wt:** 388.49; **ES-MS^+^**: 388.4.

**HPLC purity:**  The compound was found to be 98 % pure at 220 nm on analytical RP-HPLC.

**S-023-0521:N-(2-(1H-indol-3-yl)ethyl)-2-(3-oxo-3,4-dihydro-2H-benzo[b][1,4]thiazin-2-yl) acetamide**

This compound was prepared according to the general procedure, and isolated as light sand solid in 23% yield; Rf = 0.3 in EtOAc: Hex (1:1); mp: 196 ^o^C;

**1H NMR** (400 MHz, DMSO-*d6*) δ 10.80 (bs, 1H), 10.63 (s, 1H), 8.09 (s, 1H), 7.52 (d, *J* = 7.8 Hz, 1H), 7.32 (td, *J* = 8.0, 1.2 Hz, 2H), 7.23 – 7.13 (m, 2H), 7.09 – 7.03 (m, 1H), 6.99 (ddd, *J* = 7.9, 3.9, 2.5 Hz, 3H), 3.85 (dd, *J* = 9.3, 5.0 Hz, 1H), 2.82 (t, *J* = 7.3 Hz, 2H), 2.72 (dd, *J* = 15.2, 5.0 Hz, 1H), 2.32 (dd, *J* = 15.2, 9.3 Hz, 1H).

**^13^C NMR** (101 MHz, DMSO- *d6*) δ 168.56, 166.54, 137.26, 136.62, 128.14, 127.50, 123.48, 123.14, 121.27, 118.60, 118.44, 117.39, 112.11, 111.74, 81.56, 38.37, 35.53, 25.46.

**IR (CH_2_Cl_2_) *ν*** 3775.47, 3396.75, 3918.23, 1782.36, 1632.30, 1384.73 cm^-1^

**Mol wt:** 365.45; **ES-MS^+^**: 366.

**HPLC purity:**  The compound was found to be 94 % pure at 220 nm on analytical RP-HPLC.

**S-023-0522: 2-(3-oxo-3,4-dihydro-2H-benzo[b][1,4]thiazin-2-yl)-N-(thiazol-2-yl)acetamide**

This compound was prepared according to the general procedure, and isolated as light green solid in 57% yield; Rf = 0.5 in EtOAc: Hex (1:1);

**^1^H NMR** (400 MHz, DMSO- *d6*) δ 12.20 (bs, 1H), 10.70 (s, 1H), 7.47 (d, *J* = 3.6 Hz, 1H), 7.34 (dd, *J* = 8.0, 1.4 Hz, 1H), 7.25 – 7.19 (m, 2H), 7.03 – 6.98 (m, 2H), 4.00 – 3.94 (m, 1H), 3.08 (dd, *J* = 15.8, 6.2 Hz, 1H), 2.74 (dd, *J* = 15.8, 8.2 Hz, 1H).

**^13^C NMR** (101 MHz, DMSO- *d6*) δ 167.65, 165.74, 157.72, 137.63, 136.85, 127.68, 127.27, 123.14, 118.06, 117.15, 113.53, 37.38, 34.67.

**IR (CH_2_Cl_2_) *ν*** 3410.63, 2924.30, 2332.35, 1775.26, 1676.79, 1574.83 cm^-1^

**Mol wt:** 305.37; **ES-MS^+^**: 306

**HPLC purity:**  The compound was found to be 91% pure at 220 nm on analytical RP-HPLC.

**S-023-0516:N-(1H-benzo[d]imidazol-2-yl)-2-(3-oxo-3,4-dihydro-2H-benzo[b][1,4]thiazin-2-yl) acetamide**

This compound was prepared according to the general procedure, and isolated as light grey solid in 52% yield; Rf = 0.2 in EtOAc: Hex (1:1); mp: 160 ^o^C;

**^1^H NMR** (400 MHz, DMSO-*d6*) δ 10.56 (bs, 1H), 8.02 – 7.77 (m, 1H), 7.38 (ddd, *J* = 5.5, 3.4, 1.6 Hz, 2H), 7.22 (d, *J* = 7.8 Hz, 1H), 7.11 (s, 1H), 7.02 (ddd, *J* = 5.5, 3.3, 1.7 Hz, 2H), 6.99 – 6.89 (m, 2H), 3.97 (ddd, *J* = 8.0, 6.0, 1.6 Hz, 1H), 3.07 (ddd, *J* = 15.8, 6.0, 1.6 Hz, 1H), 2.80 – 2.63 (m, 1H).

**^13^C NMR** (101 MHz, DMSO-*d6*) δ 168.98, 165.85, 146.33, 136.90, 127.69, 127.28, 123.17, 121.05, 118.11, 117.18, 37.51, 35.25.

**IR (CH_2_Cl_2_) *ν*** 3775.99, 3402.32, 2921.51, 2299.78, 1643.01, 1384.63 cm^-1^

**Mol wt:** 338.3; **ES-MS^+^**: 339.09.

**HPLC purity:**  The compound was found to be 96% pure at 220 nm on analytical RP-HPLC.

**S-023-0524:N-(3,5-bis(trifluoromethyl)benzyl)-2-(3-oxo-3,4-dihydro-2H-benzo[b][1,4]thiazin-2-yl) acetamide**

This compound was prepared according to the general procedure, and isolated as white solid in 37% yield; Rf = 0.4 in EtOAc: Hex (1:1); mp: 233 ^o^C;

**^1^H NMR** (400 MHz, DMSO- *d_6_*) δ 10.66 (bs, 1H), 8.73 (t, *J* = 6.0 Hz, 1H), 7.99 (d, *J* = 5.4 Hz, 3H), 7.27 – 7.17 (m, 2H), 7.05 – 6.95 (m, 2H), 4.60 – 4.39 (m, 2H), 3.88 (dd, *J* = 9.1, 5.4 Hz, 1H), 2.84 (dd, *J* = 15.0, 5.5 Hz, 1H), 2.47 – 2.41 (m, 1H).

**^13^C NMR** (101 MHz, DMSO- *d_6_*) δ 169.56, 166.49, 143.60, 137.36, 130.78, 130.46, 128.28, 127.89, 127.70, 125.26, 123.64, 122.54, 120.96, 118.35, 117.61, 41.86, 38.52, 35.49.

**IR (CH_2_Cl_2_) *ν*** 3429.11, 3308.45, 3212.61, 2325.22, 1651.98, 1128.45 cm^-1^

**Mol wt:** 448.38; **ES-MS^+^**: 449.4.

**HPLC purity:**  The compound was found to be 100% pure at 220 nm on analytical RP-HPLC.

**S-024-1151: N-(2-fluorophenyl)-2-(3-oxo-3,4-dihydro-2H-benzo[b][1,4]thiazin-2-yl)acetamide**

This compound was prepared according to the general procedure, and isolated as white solid in 45% yield; Rf = 0.6 in EtOAc: Hex (1:1); mp: 250 ^o^C;

**^1^H NMR** (400 MHz, DMSO-*d_6_*) δ 10.69 (s, 1H), 9.87 (s, 1H), 8.05 – 7.88 (m, 1H), 7.34 (d, *J* = 7.7 Hz, 1H), 7.29 – 7.18 (m, 2H), 7.18 – 7.10 (m, 2H), 7.01 (d, *J* = 7.8 Hz, 2H), 3.93 (dd, *J* = 8.5, 5.6 Hz, 1H), 3.02 (dd, *J* = 15.6, 5.8 Hz, 1H), 2.72 (dd, *J* = 15.6, 8.5 Hz, 1H).

**^13^C NMR** (101 MHz, DMSO) δ 167.98, 165.99, 154.51, 152.07, 136.90, 127.72, 127.21, 126.07, 125.15, 124.34, 123.14, 118.09, 117.09, 115.35, 37.74, 35.62.

**IR (CH_2_Cl_2_) *ν*** 3279.95, 3199.58, 2910.92, 1663.02, 1394.57, 749.60 cm^-1^

**Mol wt:** 316.35; **ES-MS^+^**: 317.1

**HPLC purity:**  The compound was found to be 100% pure at 254 nm on analytical RP-HPLC.

**Spectral Data**

**YHV98-4: N-(4-chlorophenyl)-2-(3-oxo-3,4-dihydro-2H-benzo[b][1,4]thiazin-2-yl)acetamide**

**HPLC Chromatogram:** 97% Pure

**
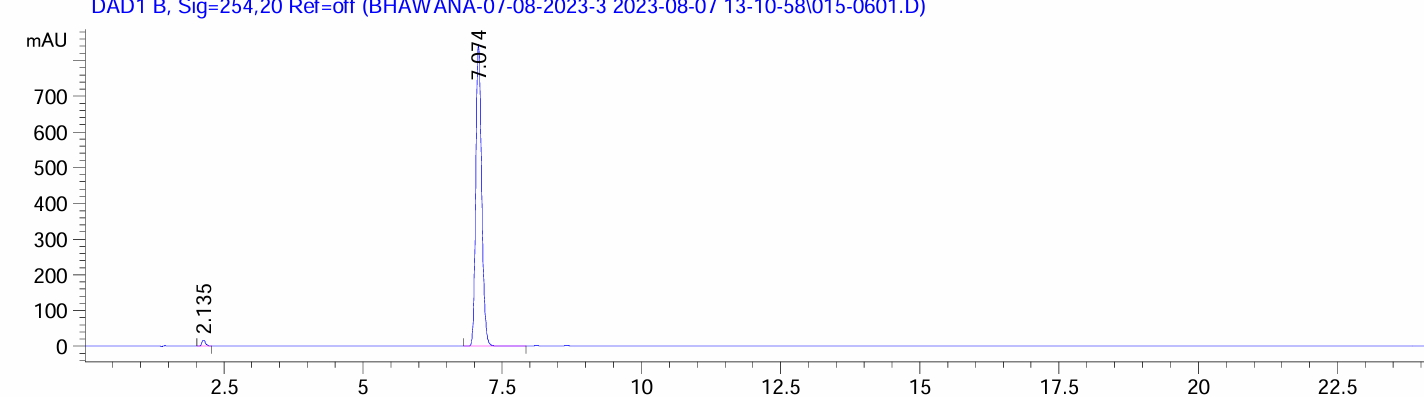
**

**S-023-0515: N-(2-chlorophenyl)-2-(3-oxo-3,4-dihydro-2H-benzo[b][1,4]thiazin-2-yl)acetamide**

**HPLC Chromatogram:** 96% Pure


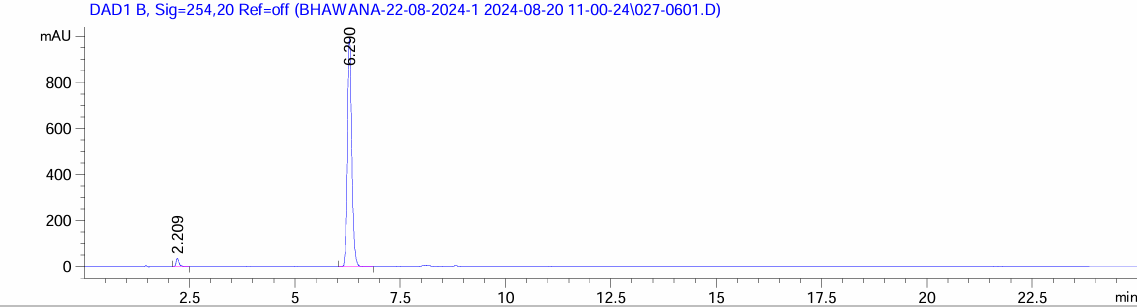


**S-023-0518: N-((3R,5S)-adamantan-1-yl)-2-(3-oxo-3,4-dihydro-2H-benzo[b][1,4]thiazin-2-yl)acetamide**

**S-023-0520: 2-(3-oxo-3,4-dihydro-2H-benzo[b][1,4]thiazin-2-yl)-N-(p-tolyl)acetamide**

**S-024-0755: N-(2-aminophenyl)-2-(3-oxo-3,4-dihydro-2H-benzo[b][1,4]thiazin-2-yl)acetamide**

**S-024-0756: N-(2-methoxyphenyl)-2-(3-oxo-3,4-dihydro-2H-benzo[b][1,4]thiazin-2-yl)acetamide**

**S-024-0752: N-(2-bromophenyl)-2-(3-oxo-3,4-dihydro-2H-benzo[b][1,4]thiazin-2-yl)acetamide**

**S-024-0754: N-(2-bromo-4-(trifluoromethoxy)phenyl)-2-(3-oxo-3,4-dihydro-2H-benzo[b][1,4] thiazin-2-yl)acetamide**

**S-024-0753: N-(2-iodophenyl)-2-(3-oxo-3,4-dihydro-2H-benzo[b][1,4]thiazin-2-yl)acetamide**

**S-024-0758: N-(2,4-dichlorophenyl)-2-(3-oxo-3,4-dihydro-2H-benzo[b][1,4]thiazin-2-yl)acetamide**

**S-023-0519: 2-(3-oxo-3,4-dihydro-2H-benzo[b][1,4]thiazin-2-yl)-N-(3-(trifluoromethyl)phenyl) acetamide**

**S-024-0757: 2-(3-oxo-3,4-dihydro-2H-benzo[b][1,4]thiazin-2-yl)-N-(1-phenylethyl)acetamide**

**S-023-0523: N-benzhydryl-2-(3-oxo-3,4-dihydro-2H-benzo[b][1,4]thiazin-2-yl)acetamide**

**S-023-0521:N-(2-(1H-indol-3-yl)ethyl)-2-(3-oxo-3,4-dihydro-2H-benzo[b][1,4]thiazin-2-yl) acetamide**

**S-023-0522: 2-(3-oxo-3,4-dihydro-2H-benzo[b][1,4]thiazin-2-yl)-N-(thiazol-2-yl)acetamide**

**S-023-0516: N-(1H-benzo[d]imidazol-2-yl)-2-(3-oxo-3,4-dihydro-2H-benzo[b][1,4]thiazin-2-yl) acetamide**

**S-023-0524: N-(3,5-bis(trifluoromethyl)benzyl)-2-(3-oxo-3,4-dihydro-2H-benzo[b][1,4]thiazin-2-yl) acetamide**

**S-024-1151:N-(2-fluorophenyl)-2-(3-oxo-3,4-dihydro-2H-benzo[b][1,4]thiazin-2-yl)acetamide**
